# Thermal Denaturation of Fresh Frozen Tissue Enhances Mass Spectrometry Detection of Peptides

**DOI:** 10.1021/acs.analchem.4c03625

**Published:** 2024-10-11

**Authors:** Angela R.S. Kruse, Audra M. Judd, Danielle B. Gutierrez, Jamie L. Allen, Martin Dufresne, Melissa A. Farrow, Alvin C. Powers, Jeremy L. Norris, Richard M. Caprioli, Jeffrey M. Spraggins

**Affiliations:** † Mass Spectrometry Research Center, Vanderbilt University, Nashville, Tennessee 37212, United States; ‡ Department of Biochemistry, Vanderbilt University, Nashville, Tennessee 37212, United States; § Department of Cell and Developmental Biology, Vanderbilt University, Nashville, Tennessee 37212, United States; ∥ Bruker Daltonics, Billerica 01821, Massachusetts United States; ⊥ Department of Medicine, Division of Diabetes, Endocrinology, and Metabolism, Vanderbilt University School of Medicine, Nashville, Tennessee 37212, United States; # VA Tennessee Valley Healthcare System, Nashville, Tennessee 37212, United States; ∇ Department of Chemistry, Vanderbilt University, Nashville, Tennessee 37212, United States; ○ Department of Medicine, Vanderbilt University, Nashville, Tennessee 37212, United States; ◆ Department of Pharmacology, Vanderbilt University, Nashville, Tennessee 37212, United States

## Abstract

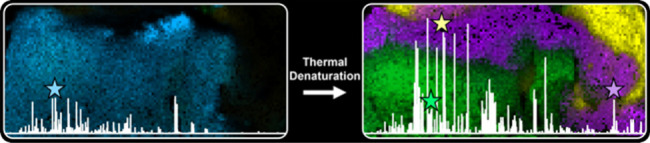

Thermal denaturation (TD), known as antigen retrieval, heats tissue samples in a buffered solution to expose protein epitopes. Thermal denaturation of formalin-fixed paraffin-embedded samples enhances on-tissue tryptic digestion, increasing peptide detection using matrix-assisted laser desorption ionization imaging mass spectrometry (MALDI IMS). We investigated the tissue-dependent effects of TD on peptide MALDI IMS and liquid chromatography-tandem mass spectrometry signal in unfixed, frozen human colon, ovary, and pancreas tissue. In a triplicate experiment using time-of-flight, orbitrap, and Fourier-transform ion cyclotron resonance mass spectrometry platforms, we found that TD had a tissue-dependent effect on peptide signal, resulting in a (22.5%) improvement in peptide detection from the colon, a (73.3%) improvement in ovary tissue, and a (96.6%) improvement in pancreas tissue. Biochemical analysis of identified peptides shows that TD facilitates identification of hydrophobic peptides.

Proteins are critical to every biological process, and their expression and regulation vary greatly by cell type and location within tissue microenvironments. Spatial mapping of proteins and peptides is crucial to understand these complex dynamics. Mass spectrometry (MS) is a powerful tool to accomplish this, as it allows for untargeted detection of proteins and the ability to map specific post-translational modifications. Spatially targeted proteomics encompasses electrospray ionization (ESI) and matrix-assisted laser desorption/ionization (MALDI) methods. ESI-based methods for spatial proteomics couple ESI to surface sampling approaches such as laser capture microdissection (LCM), liquid extraction surface analysis (LESA), and microLESA as a means to target specific tissue regions for liquid chromatography-tandem MS (LC-MS/MS).^1−4^ MALDI imaging mass spectrometry (MALDI IMS) uses a chemical matrix to facilitate extraction and ionization of surface analytes using a laser in a raster pattern.^5^ MALDI IMS enables imaging of molecular classes such as lipids, proteins, metabolites, glycans, and peptides.^6−11^ Peptide imaging involves on-tissue digestion of proteins, followed by matrix application and MALDI analysis of the resulting peptides.^12−14^ This method enables sampling of a larger subset of the proteome than can be achieved by direct analysis of intact proteins due to challenges in detecting high *m*/*z* ions via MALDI MS. However, significant improvements in signal intensity and spatial localization of proteolytic peptides are achievable through meticulous sample preparation optimization.^12,15^ Optimization of digestion efficiency is especially important for surface-sampling techniques which do not include physical disruption or detergent-based lysis steps used in liquid extractions. Here, we evaluate the effect of antigen retrieval, i.e., thermal denaturation (TD), on on-tissue protein digestion coupled to spatially targeted LC-MS/MS and MALDI IMS of fresh frozen human tissues.

Researchers have reported the application of thermal denaturation to improve the sensitivity of molecular assays in tissues.^16−19^ Thermal denaturation was first developed to enhance immunohistochemistry (IHC) in formalin-fixed paraffin-embedded (FFPE) tissue. It is also employed in flow cytometry, immunoblotting, fluorescent *in situ* hybridization, and MALDI IMS (for FFPE tissue).^14,20−24^ Researchers usually apply TD to FFPE tissues; however, it also enhances protein target detection in IHC and immunoblotting experiments in fresh frozen tissue.^16,25^ Though TD improves protein antigenicity and digestion, the mechanism is not fully understood. One proposed mechanism in FFPE tissue is the disruption of formalin-induced protein cross-links via heat or strong alkaline treatment.^20^ Thermal denaturation may also disrupt steric hindrances that limit epitope access.^25^ These effects are a plausible basis for improved on-tissue tryptic digestion in fresh frozen tissues.^15,25^

This study systematically assesses the impact of TD for on-tissue tryptic digestion and peptide analysis of tissues from human colon, ovary, and pancreas tissue. These data suggest biochemical factors underlying thermal denaturation and on-tissue enzyme digestion and how these differ by tissue source.

## Experimental Section

### Tissue Procurement

Human colonic tissue was obtained by the Cooperative Human Tissue Network from consented, deidentified donors under Institutional Review Board-approved protocol 031078. Human ovary tissue was obtained via the Henry M. Jackson Foundation for the Advancement of Military Medicine, Inc. (Award # HU00012120002). Human pancreas tissue was identified from a nondiabetic (ND), 52 year old, Caucasian male donor using a national network of partnerships, including the International Institute for Advancement of Medicine (IIAM), and acquired in partnership with the Organ Procurement and Transplantation Network (OPTN) in association with the Human Pancreas Analysis Program (HPAP) and Vanderbilt Pancreas Biorepository (VPB). The donor pancreas was processed using several fixation and preservation methods, including fresh freezing, as previously described.^26^

### Tissue Preparation and On-Tissue Digestion

Fresh frozen human pancreas, colon, and optimal cutting temperature compound (OCT) embedded ovary were prepared in triplicate for MALDI IMS with a serial section prepared for LC-MS/MS analysis. A fourth technical replicate was also prepared for MALDI Fourier transform ion cyclotron resonance (FT-ICR) IMS. Each tissue was sectioned on a cryostat (Leica Biosystems, Wetzlar, Germany) at 10 μm thickness and thaw-mounted onto indium tin oxide (ITO) coated glass slides (Delta Technologies, Loveland, Colorado). Samples were vacuum sealed and stored at −80 °C until ready for analysis. Samples were equilibrated to room temperature in the vacuum packets for 30 min after being removed from the −80 °C freezer. The OCT was removed from the ovary using a series of four 50 mM ammonium formate washes. MALDI IMS and LC-MS/MS samples were washed in Carnoy’s solution to remove salts and lipids. Briefly, slides were dipped in Coplin jars containing 70% ethanol-30 s, 100% ethanol-30 s, Carnoy’s solution-2 min, 100% ethanol-30 s, 40% ethanol-30 s, and finally, 100% ethanol-30 s. The slides were dried in the hood for 10 min. Slides were then placed in a desiccant box (standard method for fresh frozen tissue) or thermally denatured (antigen retrieval). Thermal denaturation was carried out in a digital decloaking chamber (Biocare Medical, Pacheco, California). The slides were placed in a Coplin jar containing 10 mM tris base, pH 9. The samples were heated in the chamber to 95 °C for 20 min. The chamber was then cooled to 90 °C for 10 s for safer removal of the Coplin jar. Once the jar was removed from the decloaking chamber, the slides were cooled in the buffer for 20 min prior to buffer exchange with milli-Q purified water (Millipore Sigma, Burlington, Massachusetts). Slides were placed at room temperature for approximately 5 min, then placed over desiccant to allow samples to dry. Trypsin, 3.2 ng/mm^2^ final, (Pierce porcine trypsin, MS grade) (Thermo Scientific, Waltham, Massachusetts) was applied with a modified M3 TM sprayer (HTX Technologies, LLC, Chapel Hill, North Carolina) as previously described.^12^ The slides were then preheated on a hot plate to 37 °C for 10 min to prevent condensation prior to placement in a humidity oven (ESPEC, Denver, Colorado) at 37 °C, 100% relative humidity, overnight (approximately 16 h).

### Liquid Chromatography–Mass Spectrometry

After on-tissue digestion, microextractions were collected from samples for LC-MS/MS. Briefly, 2 μL-40% acetonitrile in HPLC water containing 0.1% formic acid was pipetted up and down ten times on four locations across each tissue in a protocol adapted from previous work.^1,27−29^ The pooled extracts from each tissue were then placed in separate LoBind Eppendorf tubes containing 5 μL of 40% acetonitrile in HPLC water containing 0.1% formic acid, vortexed, spun down and dried on a Speedvac (Thermo Scientific, Waltham, Massachusetts) without heat. Samples were stored at −80 °C until ready for LC-MS/MS analysis. The protein concentration of the colon samples was assessed using a QuantiPro BCA Assay Kit (Sigma-Aldrich, St. Louis, Missouri). All extracts were desalted and concentrated using C18 EvoTips (EvoSep Biosystems, Odense, Denmark). Briefly, Evotips were washed with acetonitrile in HPLC water containing 0.1% formic acid (Solvent B), conditioned in 1-propanol, and equilibrated with HPLC water containing 0.1% formic acid (Solvent A). The pancreas and ovary samples were reconstituted in 20 μL of Solvent A and added to the prepared Evotips. Since the colon samples were quantified using the QuantiPro BCA assay, the appropriate volume of sample to equal 500 ng protein was added to the prepared Evotip, and Solvent A was added to equal 20 μL total volume. Samples were washed with Solvent A and then preserved in 100 μL of Solvent A. The samples were stored at +4 °C until analysis. Liquid chromatography–mass spectrometry was performed using an EvoSepOne (EvoSep Biosystems, Odense, Denmark). Analytical separation was performed using a 15 cm long, 150 μm inner diameter column containing Dr Maisch C18 AQ resin with 1.9 μm beads and a fused silica emitter tip (20 μm inner diameter) (EvoSep). Peptides were eluted from the column using an EvoSep 15 samples/day method. Mass spectrometry was performed on an Orbitrap Fusion (ThermoFisher Scientific) equipped with a Nanospray Flex Ion source. Data-dependent analysis was performed, selecting the top 12 most abundant precursors per scan for HCD fragmentation. Orbitrap detection was performed with a resolution of 60,000 at *m*/*z* 200 with a normalized AGC target rate of 250%.

### Proteomics Data Analysis

Raw proteomics data files were searched against a database containing human proteins along with common contaminants using MaxQuant.^30^ Search parameters allowed for two missed tryptic cleavages, variable N-terminal acetylation, methionine oxidation, and proline oxidation. Separate database searches allowed for the variable modifications above along with serine, threonine, and tyrosine phosphorylation, and tyrosine sulfation. Protein identifications were made with a minimum of two razor + unique peptide matches. Label-free quantitation was performed using MaxQuant, and a quality control assessment of the searched data was done using Proteomics Quality Control (PTXQC).^31^ Treatment groups were statistically compared using a Welch’s test with a P-value cutoff of 0.05, and results were visualized using Perseus.^32^ Gene ontology analysis was performed using Panther^33^ and Rstudio^34^ was used for data visualization. All mass spectrometry proteomics data have been deposited to the ProteomeXchange Consortium via the PRIDE partner repository with the data set identifier PXD048978.^35^

### MALDI IMS

MALDI IMS samples were coated with a MALDI matrix, as previously described.^12^ Briefly, 5 mg/mL α-cyano 4-hydroxycinnamic acid in 90% acetonitrile in 0.1% trifluoroacetic acid was applied using an M3 Sprayer (HTX Technologies, Chapel Hill, NC). Samples were stored out of light and over desiccant until ready for analysis. MALDI IMS was performed on technical replicates using a Bruker ultrafleXtreme time-of-flight mass spectrometer (Bruker, Billerica, Massachusetts) in reflectron positive ion mode using 100 shots per pixel with 100 μm lateral resolution, no random walk, and an *m*/*z* range from 600 to 4,497. Each imaging region consisted of approximately 5,000, 4,000, and 3,000 pixels for the colon, ovary, and pancreas, respectively. Supporting high mass accuracy data were collected from a single serial tissue section using a Bruker SolariX 15T FT-ICR MS (Bruker, Billerica, Massachusetts) with 100 μm lateral resolution in positive ion mode, 200 shots per pixel with no random walk, and an *m*/*z* range of 346–3,000. Each FT-ICR imaging region consisted of approximately 500,000 pixels.

### Stained Microscopy

After MALDI IMS, the matrix was removed from all samples using incubation in 90% and 70% ethanol for 30 s each until no matrix was visible. Samples were then hematoxylin and eosin stained using the following series of solutions: deionized water, hematoxylin, deionized water, 0.5% ammonium hydroxide, deionized water, 70% ethanol, 95% ethanol, eosin, 95% ethanol, 100% ethanol, and xylenes. Coverslips were applied to samples using cytoseal coverslipping medium. Stained tissues were imaged using a Leica SCN 400 slide scanner using a 20× magnification objective (Leica Biosystems, Wetzlar, Germany).

## Results and Discussion

**Figure 1:**
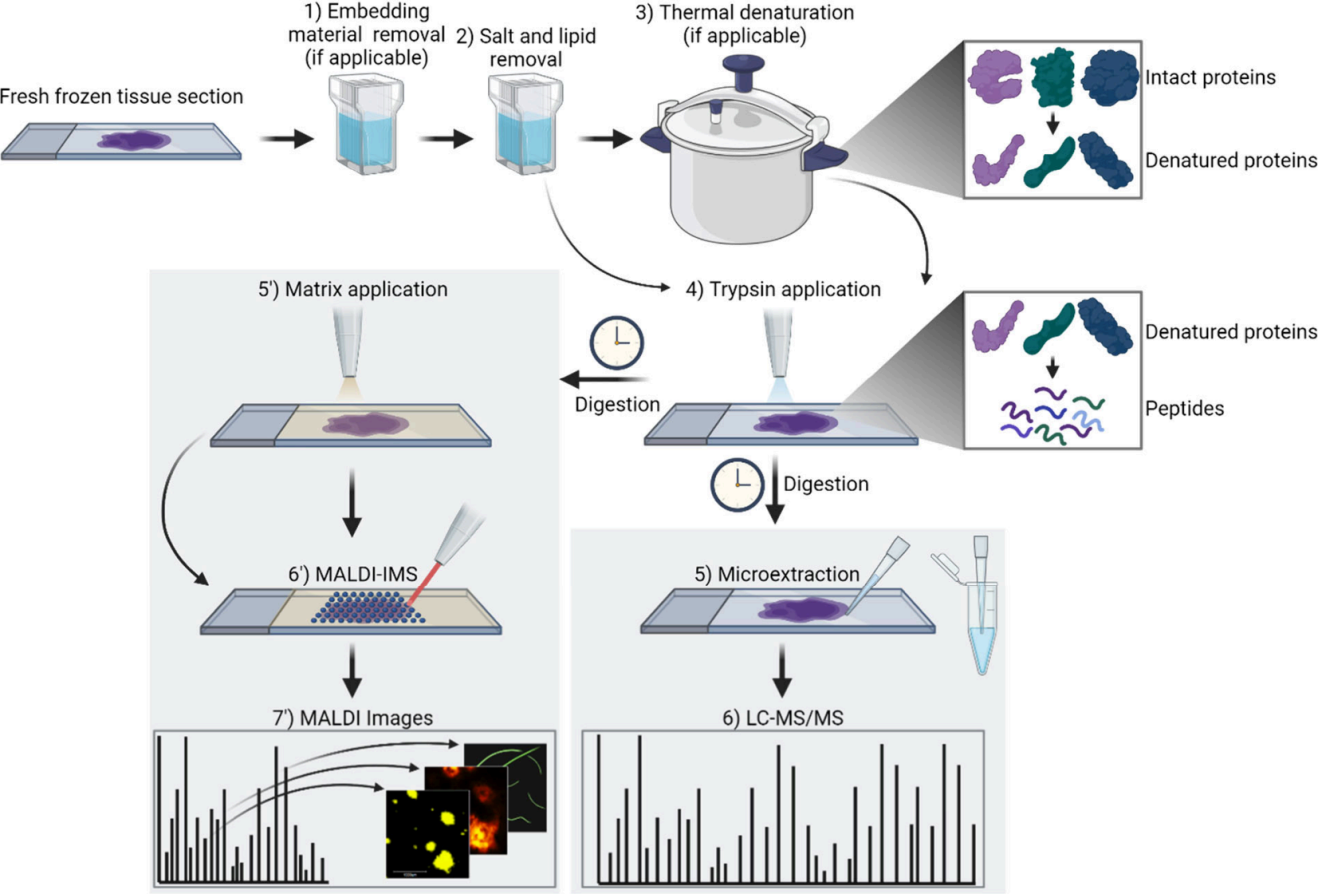
Experimental workflow. Fresh frozen tissue sections are subjected to washing steps and optional thermal denaturation to improve digestion efficiency. On-tissue trypsin digestion produces tryptic peptides. Samples can then be coated with a matrix to assist with the extraction and ionization of peptides, followed by matrix-assisted laser desorption ionization imaging mass spectrometry (MALDI IMS). In tandem, microextraction can extract surface peptides for analysis via liquid chromatography-tandem mass spectrometry (LC-MS/MS).

To systematically investigate the effects of TD on fresh frozen tissue for LC and MALDI IMS, we used a workflow based on that used for FFPE tissue (Figure 1).^12^ Fresh frozen tissue sections were mounted onto ITO-coated slides and washed to remove embedding material, salts, and lipids. Next, half of the sample slides underwent thermal denaturation in an aqueous tris buffer with an alkaline pH. Tissues were sprayed with a homogeneous coating of trypsin, and proteins were digested for 16 h in an incubator maintained at 100% relative humidity. The effect of TD on peptide identification was assessed using LC-MS/MS analysis of microextractions from the tissue surface. The effects of TD on peptide detection and localization for IMS were evaluated by applying CHCA to the tissue surface, followed by MALDI IMS using either a linear TOF or an FT-ICR mass spectrometer.

## Thermal Denaturation Enhances LC-MS/MS Protein and Peptide Identification

**Figure 2:**
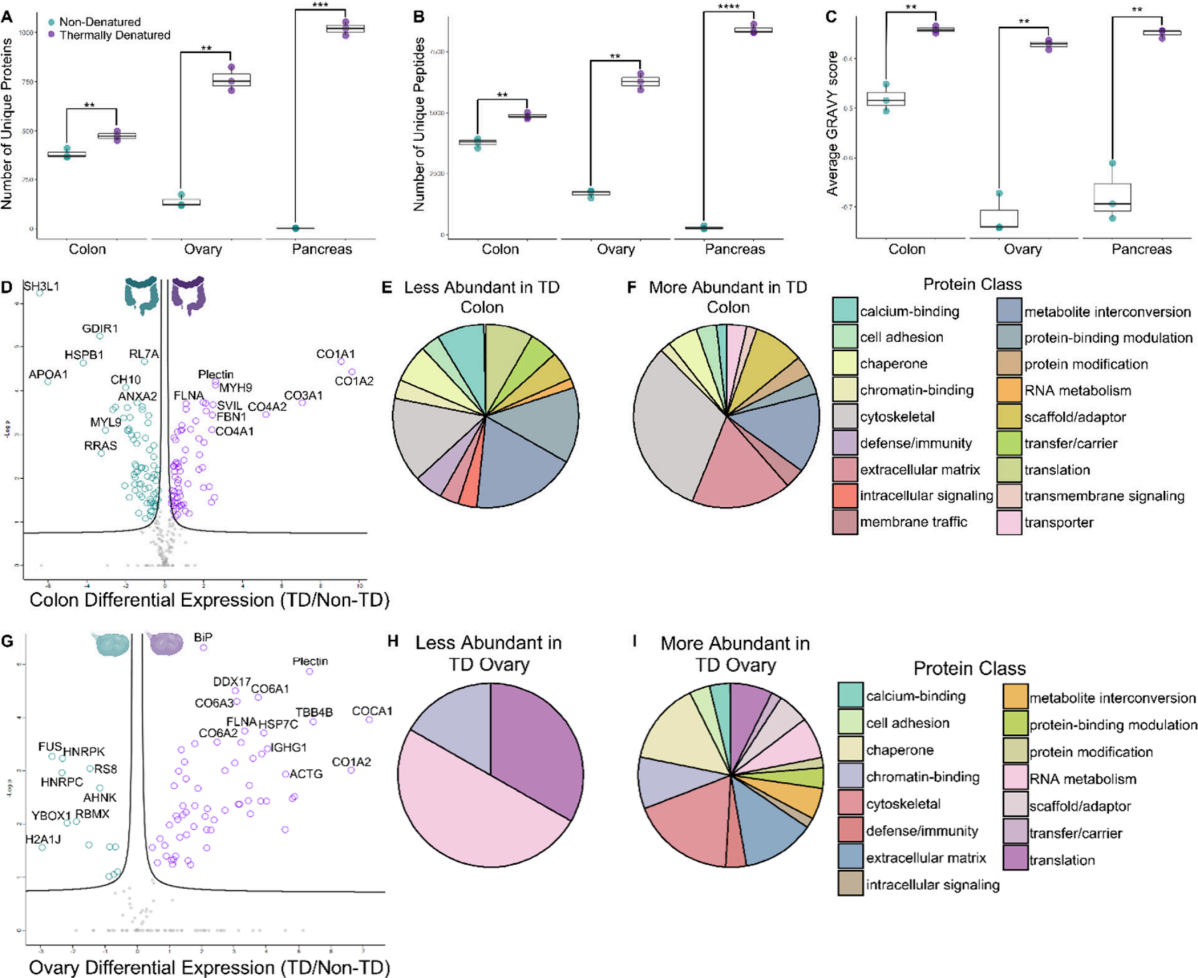
Summary and differential abundance analysis of LC-MS/MS data from microextractions. A. The number of unique proteins identified in colon, ovary, and pancreas samples without thermal denaturation (blue) and with thermal denaturation (purple). B. The number of unique peptides identified in colon, ovary, and pancreas samples without thermal denaturation (blue) and with thermal denaturation (purple). C. The Global Average of Hydropathy (GRAVY) value for identified peptides from nondenatured (blue) and thermally denatured (purple) samples in the colon, ovary, and pancreas in which a higher GRAVY score indicates higher average hydrophobicity. Student’s T-Test result **** *P* < 0.05, ** *P* < 0.01, *** *P* < 0.001, **** *P* < 0.0001. Differential abundance analysis of LC-MS/MS data using label-free quantitation comparing nondenatured and thermally denatured (TD) colon (D,E,F) and ovary (G,H,I). Pancreas tissue was omitted from this analysis because nondenatured pancreas tissue had insufficient protein identifications for statistical comparison. D. Volcano plot visualizing proteins more abundant (in purple) and less abundant (in blue) in TD colon. Proteins with increased abundance in TD tissue included Collagens alpha-1(I), alpha-2(I), alpha-1(III), alpha-1(IV), and alpha-2(IV) (CO1A1, CO1A2, CO3A1, CO4A1, CO4A2), Plectin, Myosin-9 (MYH9), Filamin-A (FLNA), Supervillin (SVIL), and Fibrillin-1 (FBN1). Proteins with decreased abundance in the TD colon include SH3 domain-binding glutamic acid-rich-like protein (SH3L1), Rho GDP-dissociation inhibitor 1 (GDIR1), Heat shock protein beta-1 (HSPB1), 60S ribosomal protein L7a (RL7A), Apolipoprotein A-I (APOA1), 10 kDa heat shock protein (CH10, HSPE1), Annexin A2 (ANXA2), Myosin regulatory light polypeptide 9 (MYL9), Ras-related protein R-Ras (RRAS). E. Gene ontology (GO) analysis representing the protein class of proteins less abundant in TD colon. F. GO analysis of proteins more abundant in TD colon. G. Volcano plot visualizing proteins more abundant (in purple) and less abundant (in blue) in TD ovary. Proteins more abundant in TD ovary include Endoplasmic reticulum chaperone BiP (BiP), Plectin, Probable ATP-dependent RNA helicase (DDX17), collagens alpha-1(VI), alpha-2(VI), alpha-3(VI), alpha-1(XII), alpha-2(I), (CO6A1, CO6A2, CO6A3, COCA1, CO1A2), Tubulin beta-4B chain (TBB4B), Filamin-A (FLNA), Heat shock cognate 71 kDa protein (HSP7C), Immunoglobulin heavy constant gamma 1 (IGHG1), and Actin (ACTG). Proteins less abundant in TD ovary include RNA-binding protein FUS (FUS), Heterogeneous nuclear ribonucleoprotein K (HNRPK), 40S ribosomal protein S8 (RS8), Neuroblast differentiation-associated protein (AHNK), Y-box-binding protein 1 (YBOX1), RNA-binding motif protein (RBMX), Histone H2A type 1-J (H2A1J), Heterogeneous nuclear ribonucleoproteins C1/C2 (HNRPC). H. GO classes of proteins less abundant in TD ovary. I. Classes of proteins more abundant in TD ovary.

The average number of unique peptides and protein groups identified by LC-MS/MS among three biological replicates per tissue were used to assess the effect of TD on peptide digestion and depth of molecular coverage. Protein group identifications were filtered to require a minimum of two unique peptide matches. In colon tissue, the average number of identified peptides increased from 3,771 to 4,867 in thermally denatured tissues, and the number of identified protein groups increased from 382 to 474 (Figure 2A-B, Table 1, Table S1). Ovary tissue showed further improvement, with the number of identified peptides increasing from 1,676 to 6,266. Thermal denaturation led to the identification of 760 protein groups, whereas only 136 protein groups were identified without TD (Figure 2A-B, Table 1). The pancreas produces many proteolytic enzymes, making it uniquely challenging for proteomic studies, often resulting in diminished peptide and protein signals in LC and imaging workflows.^36^ The pancreas analysis showed the most dramatic improvement in peptides and proteins detected using TD. The number of identified peptides and proteins increased from 286 to 8,397 and 2 to 1,020, respectively (Figure 2A-B Table 1). Based upon these metrics in a triplicate experiment, TD resulted in a 22.5% increase in peptide detection from the colon, a 73.3% increase for ovary tissue, and a 96.6% increase for pancreas tissue (Table 1).

**Table 1:** Summary of LC-MS/MS proteomics results by tissue and TD

Tissue Type	TD status	Average unique peptides ± standard deviation (*n* = 3)	Average unique protein ± standard deviation (*n* = 3)
Colon	Non-TD	3771.7 ± 157.33	382.3 ± 20.53
	TD	4867.3 ± 106.28	474.3 ± 19.62
Ovary	Non-TD	1676.3 ± 131.52	136.7 ± 25.77
	TD	6266.7 ± 271.13	760.3 ± 48.94
Pancreas	Non-TD	286.7 ± 57.13	2.0 ± 0.82
	TD	8397.0 ± 153.23	1020.0 ± 28.18

To determine whether TD enhanced the identification of hydrophobic peptides, we calculated the Global Average of Hydropathy (GRAVY) scores for peptides identified in each sample (Figure 2C). There was a statistically significant increase in the average hydrophobicity of peptides identified in TD samples. This increase was observed in colon, ovary, and pancreas tissue. These data are consistent with a proposed mechanism for TD in which protein denaturation exposes hydrophobic regions for digestion and subsequent detection via LC-MS/MS. A comparison of the relative abundance of each individual amino acid supports this interpretation, showing an overall decrease in the representation of hydrophilic residues and an increase in neutral and hydrophobic residues (Figure S1). TD in the colon resulted in a relative decrease in hydrophilic amino acids such as aspartate and glutamine, indicating that hydrophilic peptides comprise a smaller percentage of the extracted peptide population. (Figure S1A). In a triplicate comparison in colon, the average relative abundance of lysine and arginine residues decreased from 5.2 ± 0.05% to 4.7 ± 0.11% and 4.1 ± 0.05% to 3.8 ± 0.07%, respectively, suggesting an improvement in trypsin digestion efficiency (Figure S1A). This trend was also observed in the ovary with a decreased relative abundance from 5.6 ± 0.11% to 4.5 ± 0.03% and 4.5 ± 0.02% to 3.8 ± 0.02% for lysine and arginine, respectively.

Interestingly, ovary tissue showed the most dramatic increase in digestion efficiency based upon the average number of missed tryptic cleavages (Table S2) and missed cleavage metric generated using Proteomics Quality Control (PTXQC).^34^ Neutral and hydrophobic amino acids such as proline, glycine, and alanine were also shown to increase in TD samples. The same trend was seen in peptides identified in ovary tissue, with a decreased proportion of hydrophilic amino acids after TD but an increase in the proportion of neutral and hydrophobic amino acids, particularly leucine and glycine (Figure S1B). A much higher proportion of proline-containing peptides were identified in non-TD pancreas (Figure S1C). This likely results from abundant and proline-rich collagen peptides that dominated the proteomics sample while other low-abundance proteins were not effectively digested. TD pancreas tissue was found to have a dramatically higher proportion of leucine residues, with the identification of proteins including leucine-rich repeat-containing protein 59 (LRC59) (Figure S1C, Table S1). These data indicate that small, water-soluble proteins may be removed or delocalized during TD, and studies focused on these analytes may forego use of TD. Our results also suggest that TD-induced protein unfolding allows for improved digestion and detection of hydrophobic and neutral proteins, and studies focused on these targets, e.g., collagen or leucine-rich proteins, may benefit from TD. Finally, our results indicate that digestion efficiency increases after TD, especially in tissues such as the ovary and pancreas.

To assess the effect of TD on the identification of post-translational modifications, we performed database searches of the LC-MS data, allowing for variable serine, threonine, and tyrosine phosphorylation, as well as tyrosine sulfation. Colon and ovary samples were included in this analysis, and pancreas samples were excluded because the low number of peptide identifications in non-TD pancreas samples precluded statistical comparison with TD samples. Of the 85 phosphorylated proteins identified in colon samples, 31 were only detected in TD samples, and 16 were only detected in non-TD samples (Table S3). Of the phosphorylated proteins detected in both groups, five had statistically higher intensities in non-TD colon tissue, and 16 proteins were more abundant in TD colon tissue (Figure S2A,B). In ovary tissue, 32 phosphorylated proteins were identified (Table S3), of which five were only detected in non-TD samples (Figure S2C), two were statistically more abundant in TD samples (Figure S2D), and 16 were only detected in TD samples (Figure S2E). Based on these data, TD resulted in a 55% and 56% improvement in the detection of phosphorylated proteins in the colon and ovary, respectively.

TD also enhanced the identification of tyrosine sulfation. In colon tissue, 11 sulfated proteins were identified (Table S4, Figure S3A). Five of these were detected only in TD samples (FGF2, FKBP4, HUWE1, RHG05) and one in non-TD samples (IPYR). Two had higher statistical abundance in TD samples (SYNEM, SYNP2). Five sulfated proteins were identified in ovary samples, and all of these were only identified in TD samples (Figure S3B). These results indicate that TD facilitates the identification of protein modifications, which may relate to modification-induced changes to overall protein hydrophobicity.^37^

## Thermal Denaturation Impacts Protein Class Representation

Differential expression analysis compared the abundance of proteins identified in common in both TD and non-TD colon and ovary tissue. Notably, this analysis does not include proteins only detected in TD tissues. Additionally, pancreatic tissue was excluded from this analysis based on the low number of protein identifications in the non-TD pancreatic tissues (2) relative to the TD pancreatic tissues (1,020). Based upon Welch’s T-Test using a significance cutoff of 0.05, an average of 66 proteins were more abundant in TD colon tissue, and 75 proteins were less abundant (Figure 2D, Table S1). Collagen proteins (CO1A1, CO1A2, CO3A1, CO4A1, CO4A2), myosin (MYH9), plectin, fibronectin (FBN1), and filamin A (FLNA) were among the proteins with significantly increased abundance in TD colon. Apolipoprotein (APOA1), heat shock protein B1 (HSPB1), rho GDP-dissociation inhibitor 1 (GDIR1), and 60S ribosomal protein L7a (RL7A) were among the proteins with decreased abundance in the TD colon. Gene ontology (GO) analysis was used to characterize the protein classes of these differentially expressed proteins (Figure 2E,F). Cytoskeletal, extracellular matrix, and membrane trafficking proteins were more abundant in TD colon tissue (Figure 2F). Protein-binding modulator, intracellular signaling, and RNA metabolism proteins were less abundant in the TD colon tissue (Figure 2E). These results are more nuanced in the colon compared to the ovary. In the ovary, an average of 63 proteins were statistically more abundant in TD tissue, and 13 proteins were less abundant. Cytochrome c oxidase (COCA1), collagen (CO1A2), tubulin beta-4 chain (TBB4B), and ATP-dependent RNA helicase (DDX17) were among the proteins with increased abundance, and RNA-binding protein FUS (FUS), Heterogeneous nuclear ribonucleoproteins (HNRPK and HNRPC), 40S ribosomal protein (RS8), and neuroblast differentiation-associated protein AHNAK (AHNK) were among those with decreased abundance in TD ovary (Figure 2G). GO analysis to classify these proteins revealed an overall increase in protein class diversity in TD ovary tissue, with enhanced identification of cell adhesion, chaperone, metabolite interconversion, and cytoskeletal proteins (Figure 2H–I). Proteins more abundant in non-TD ovary tissue were classified as RNA metabolism, chromatin binding, and translational proteins (Figure 2I). Overall, each tissue showed increased diversity of protein class identified after TD. Specifically, TD led to improved identification of extracellular matrix and cytoskeletal proteins. As these proteins may differ in biochemical property and hydrophobicity, it is likely that protein unfolding and subsequent improved digestion allowed for the identification of these classes.

## Thermal Denaturation Improves the Detection of Peptides Via MALDI IMS

**Figure 3:**
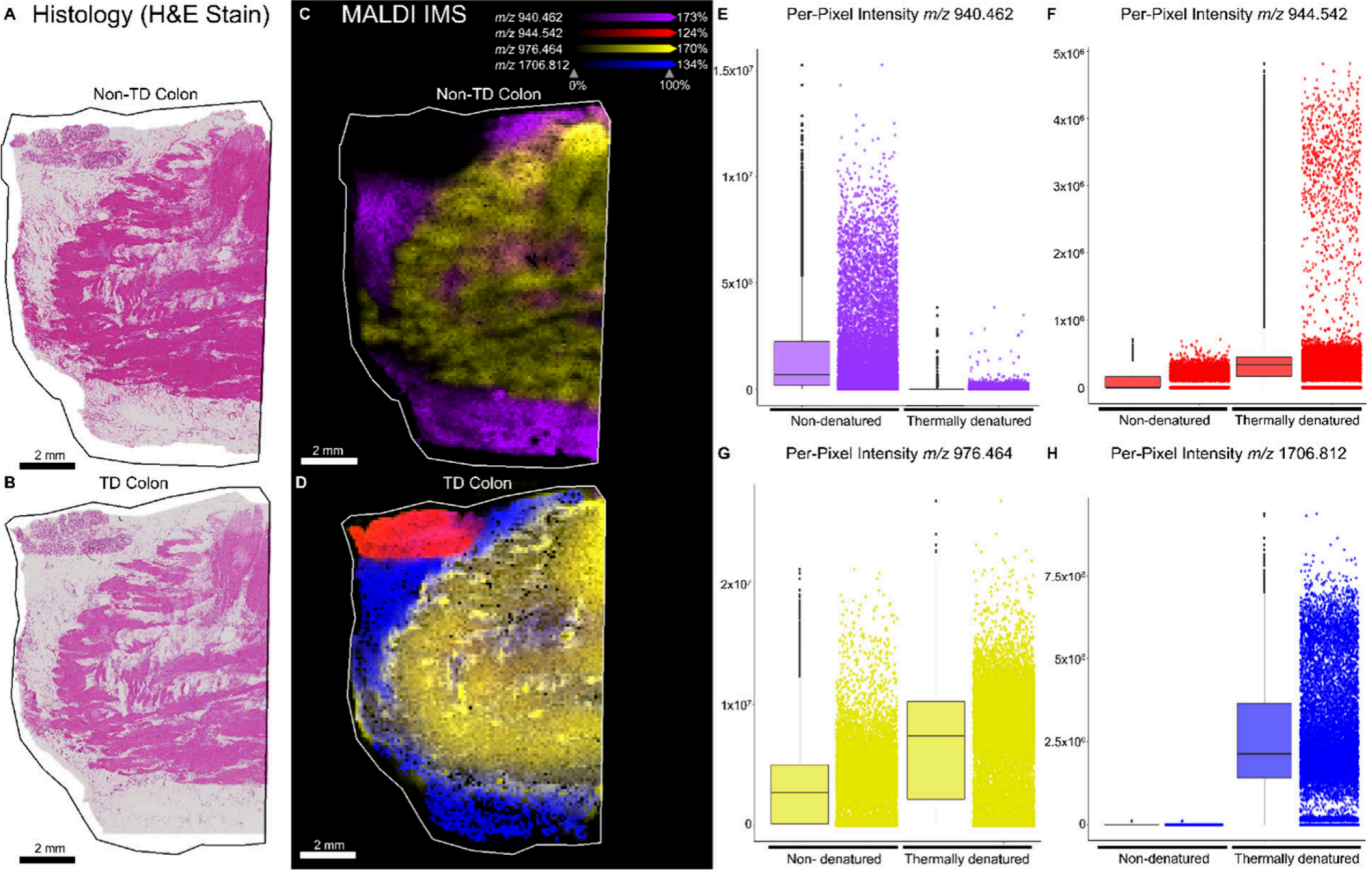
Post-MALDI IMS H&E stain of serial sections of nondenatured (A) compared to thermally denatured (B) human colon tissue. Ion images of peptides with *m*/*z* values 940.4621 (purple), 944.5420 (red), 976.4640 (yellow), and 1706.8124 (blue) in nondenatured (C) and TD (D) tissue. (E) Ion intensity plot of *m*/*z* 940.4621 shows increased detection in nondenatured tissue. (F) Ion intensity plot of *m*/*z* 944.5420 shows increased detection in TD tissue. (G) Ion intensity plot of *m*/*z* 976.4640 shows moderate increase in detection in TD tissue. (H) Ion intensity plot of *m*/*z* 1706.8124 showing dramatically improved detection in TD tissue.

**Figure 4:**
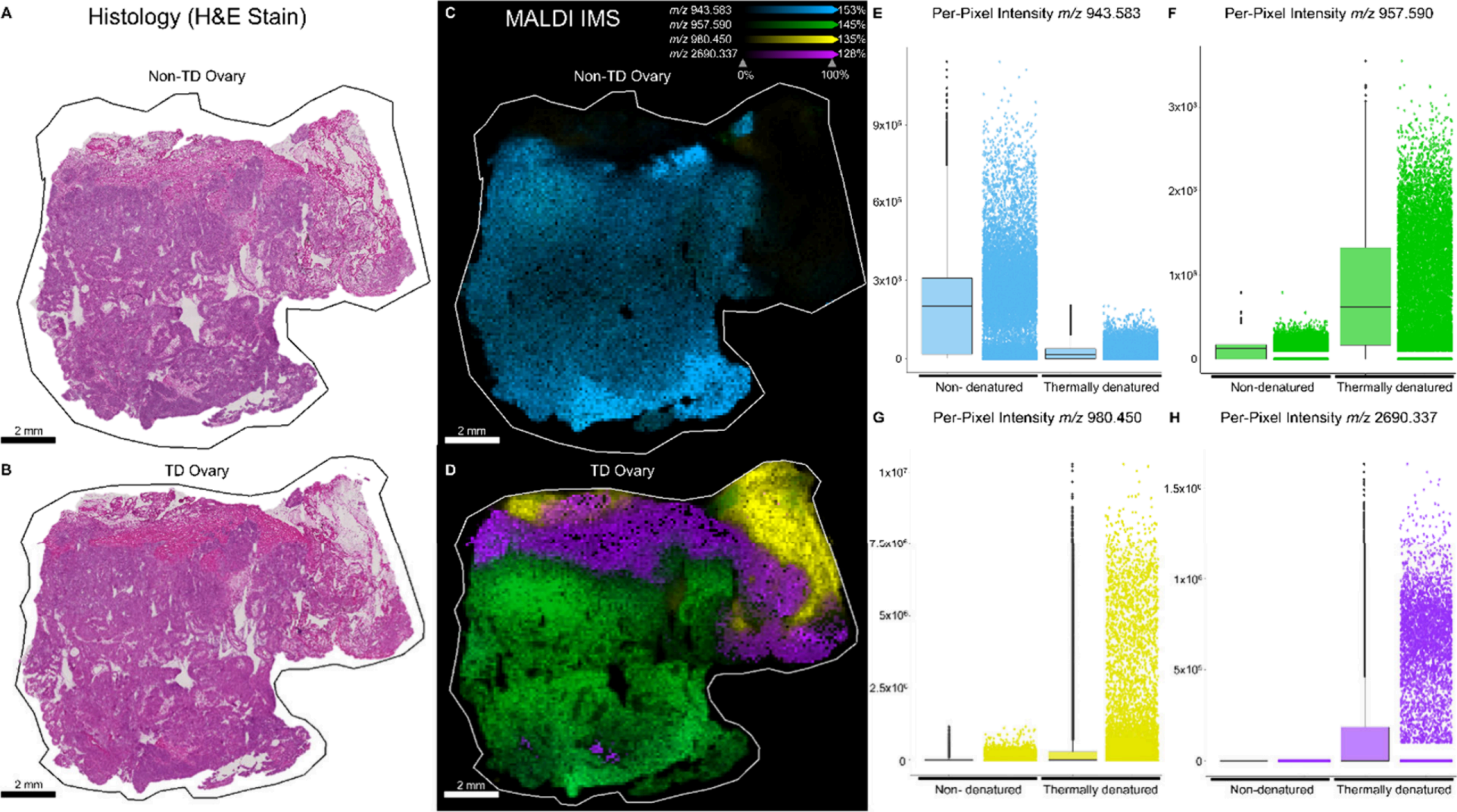
Post-MALDI IMS H&E stain of serial sections of nondenatured (A) compared to thermally denatured (B) human ovary tissue. Ion images of peptides with *m*/*z* values 943.583 (blue), 957.590 (green), 980.450 (yellow), and 2790.337 (purple) in nondenatured (C) and TD (D) tissue. (E) Ion intensity plot of *m*/*z* 943.583 shows increased detection in nondenatured tissue. (F) Ion intensity plot of *m*/*z* 957.590 shows increased detection in TD tissue. (G) Ion intensity plot of *m*/*z* 980.450 shows increased detection TD tissue. (H) Ion intensity plot of *m*/*z* 2790.337 showing increased detection in TD tissue.

The impact of TD on the MALDI IMS signal was assessed on serial tissue sections of each tissue type using both a linear TOF and FT-ICR MS. TOF measurements were used to qualitatively evaluate the molecular coverage of the TD and non-TD workflows and highlight the reproducibility of the methods in a triplicate experiment (Figures S4–S9). To evaluate spectral and imaging differences with higher mass resolution, we conducted MALDI IMS of complete tissue sections with and without TD using FT-ICR IMS. Like the linear TOF experiment, the averaged MALDI FT-ICR IMS spectra from the TD colon, ovary, and pancreas tissues are richer and more complex than non-TD tissues, particularly above *m*/*z* 1,000 (Figure S10, Table 2). Consistent with the LC-MS/MS results, the colon shows improvement in peptide detection and localization after thermal denaturation (Figure S10A,B, Figure 3). Ovary tissue shows a more dramatic improvement in overall spectral complexity compared to nondenatured (Figure S10C,D, Figure 4), and the pancreas shows a significant improvement in the overall peptide signals detected (Figure S10E,F, Figure S11).

**Table 2:** Summary of *m/z* features in FTICR MALDI IMS spectra

Tissue Type	TD status	Number of *m*/*z* features (*n* = 1)
Colon	Non-TD	310
	TD	866
Ovary	Non-TD	478
	TD	1156
Pancreas	Non-TD	60
	TD	660

In colon, some ions are more abundant or only detected above background in TD tissues (Table S5, e.g., *m*/*z* 836.445, 1094.607, 1561.815, 2057.014, 2115.162, 2705.296) and others are more abundant or exclusively above background in non-TD tissues (Table S5, e.g., *m*/*z* 848.398, 701.327, 940.461, 949.473). Some are abundant in both treatment types (Table S5, e.g., *m*/*z* 1198.721, 1790.921). We extracted and manually inspected all *m*/*z* features above a 5% intensity threshold relative to base peak for each tissue type to assess global ion detection and localization differences. Of the 114 ions above this threshold in colon tissue, 19% were detected and shared a similar localization in non-TD and TD tissue; 75% were detected in TD tissues but not above background levels in non-TD tissue, and 5% were detected more abundantly in non-TD tissue. To highlight these differences, we selected four ions with differing intensities and localization in the colon (Figure 3). Post-IMS hematoxylin and eosin (H&E) staining shows that each colon tissue section contains muscular and mucosal regions and indicates no major adherence issues during thermal denaturation (Figure 3A,B). The ion at *m*/*z* 940.462 was more abundant in the non-TD colon, as shown in the ion images (Figure 3C–D) and individual pixel intensities (Figure 3E). The ion at *m*/*z* 976.464 was detected at comparable levels in TD and non-TD colon (Figure 3C,D,G), whereas ions at *m/z’s* 944.542 and 1706.812 were more abundant in TD colon (Figure 3D, F, H).

Post-IMS histological (H&E) staining of ovarian tissue reveals no discernible adherence issues during TD and shows that the tissue samples have at least three distinct histological regions (Figure 4A-B). We manually inspected the localizations of 242 ions in ovary tissue, and 33% were comparably detected in non-TD and TD tissue, 47% were more abundantly detected in TD tissue, and 19% were more abundant in non-TD tissue (Table S6). Four ions were selected to represent these differences, and of these, *m*/*z* 943.583 was more abundant in the non-TD ovary (Figure 4C-E), whereas *m/z’s* 957.590, 980.450, and 2690.337 were detected more abundantly in TD ovary based on ion image localization (Figure 4D) and per-pixel ion intensity (Figure 4F–H).

Finally, though the overall peptide signal increased most dramatically in pancreas tissue, post-IMS H&E staining reveals poor tissue adherence during TD (Figure S11A,B). Despite this, all three representative ions (*m/z’s* 1032.609, 2125.136, and 2728.388) were detected most abundantly in TD pancreas tissue based on ion images and per-pixel intensity (Figure S11C-G). Furthermore, 80% of the ions manually inspected are only detected above background in TD pancreas (Table S7). Pancreatic tissue likely exhibits poor adhesion to the hydrophobic surface of ITO slides because this tissue has a high lipid content and is, therefore, more inherently hydrophobic than other tissues. Poly lysine coated ITO slides provide a positively charged surface to interact with the tissue and significantly improved adhesion during TD (Figure S12). Future studies can evaluate this and other solutions for improved adhesion, and this strategy is recommended for future pancreas peptide IMS workflows.

## Localization Vs Signal Intensity

**Figure 5:**
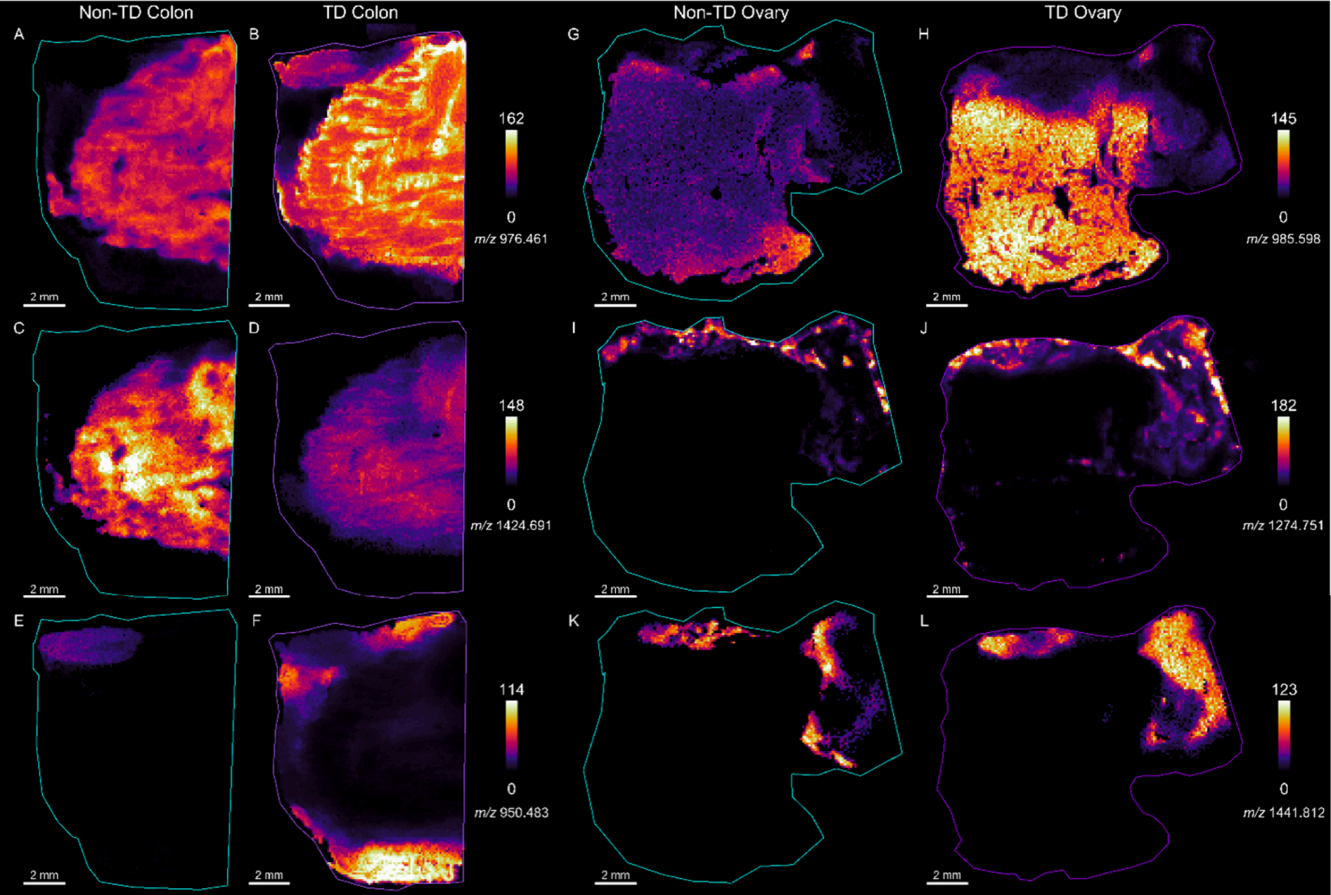
MALDI IMS ion images from nonthermally denatured (A,C,E) compared to thermally denatured (B,D,F) colon tissue and nonthermally denatured (G,I,K) compared to thermally denatured (H,J,L) ovary.

A trade-off exists between increased signal intensity and delocalization of select analytes when thermally denaturing fresh frozen tissue. Based upon the LC-MS and subsequent GRAVY analysis of microextraction samples, hydrophilic analytes may be more susceptible to delocalization during TD, given that this treatment enhances the detection of hydrophobic peptides (Figure 2C). This effect includes intratissue and extra-tissue delocalization. To assess intratissue delocalization, we manually inspected the localizations of ions in TD and non-TD tissues. Select ions are detected in different tissue regions after TD (Figure 5, Table S5). For example, *m*/*z* 976.461 is detected in the muscular region of the non-TD colon (Figure 5A), but after TD, this ion is detected in both the muscular and mucosal regions (Figure 5B). This effect may be because of improved digestion efficiency, increasing the sensitivity, allowing for a more accurate representation of this peptide’s distribution. Alternatively, TD may reveal additional isobaric peptides that complicate the ion images. Future work can explore increased mass resolution and ion mobility separation to separate isobaric peptides in IMS. Of the ions manually inspected, only one feature (*m*/*z* 2088.136) shows clear delocalization in TD colon tissue. Of the manually inspected ions in ovary and pancreas tissue, two are delocalized in TD ovary tissue (*m/z’s* 1165.569, 1441.812) (Figure 5), and one is delocalized in TD pancreas (*m*/*z* 852.441) (Figure S13). Most ions with differing localizations in TD tissue share a similar localization but appear to have delocalized slightly within tissue during TD (Figure 5J,L). Based upon our observations of increased identification of hydrophobic peptides after TD, it is likely that smaller hydrophilic proteins are more susceptible to this delocalization. Future work can explore the impact of buffer composition, e.g., acidic compared to basic pH, on peptide identification.^13^

Measurement of regions immediately outside of the tissue border was used to estimate the degree of extra-tissue delocalization. This spectrum was moderately more complex adjacent to the TD colon tissue, comparable in the TD ovary, and more complex in the TD pancreas (Figure S14). Solely based on spectral complexity, this result implies that pancreas tissue is most susceptible to extra-tissue delocalization. However, this may be acceptable given the significant increase in signal intensity in the TD pancreas compared to the non-TD pancreas. Future work can assess the effects of TD temperature and buffer formulations to minimize intra- and extra-tissue delocalization for high-resolution IMS. Furthermore, analyte delocalization in IMS has been shown to vary by analyte, tissue type, and sample preparation and is typically assessed via manual inspection of ion intensity and localization.^38,39^ Future work may establish methods to quantify the degree of intra- and extra- tissue delocalization to make this process less subjective and more efficient.

## Conclusions

Mass spectrometry-based proteomics is key to the continued study of biological systems, with spatial proteomics providing an additional context for interactions between and among tissue structures.^1,4,40−42^ Previous studies applied thermal denaturation to fresh frozen tissues to increase the sensitivity of several biochemical assays of proteins, but the mechanisms that lead to this improvement are not fully understood.^15,19^ Our study demonstrates that TD improves the identification of peptides via LC-MS/MS and MALDI IMS in human colon, ovary, and pancreas. We observed these improvements using two different mass spectrometry workflows with different ionization strategies and spatial resolutions. Though TD is known to improve trypsin digestion for MALDI IMS, a thorough comparison of its effects on different human tissues has not been done.^11,14^ Here, we show that TD improves on-tissue tryptic digestion for mass spectrometry analysis in a tissue-dependent manner.

The colon showed a small improvement in peptide identification and imaging. The ovary showed a significant improvement in most protein classes. The pancreas showed an even greater improvement after thermal denaturation.

Our findings argue for using TD for bottom-up spatial proteomics experiments with customized method optimization for new target tissues. These results likely extend to other workflows involving on-tissue enzyme applications such as PNGase and subsequent glycan analysis;^11,43−45^ however, further studies are needed to optimize TD for these applications. This work indicates that the optimal sample preparation may vary by sample source and application. For example, a researcher focused on collagen or calcium-binding proteins may forego thermal denaturation, whereas one interested in membrane proteins may choose to thermally denature fresh frozen tissues (Figure 2F). These results strongly argue for individualized method development by tissue source.

## Supporting Information Available

Table S2: PTXQC missed cleavage report. Figure S1: Amino acid composition of LC-MS identified peptides from microextraction samples from non-TD compared to TD tissues. Figure S2: Identification of proteins containing phosphorylated serine, threonine, or tyrosine posttranslational modifications in colon and ovary. Figure S3: Proteins with tyrosine sulfation detected in colon and ovary. Figure S4: Average ultrafleXtreme MALDI IMS spectra from non-TD compared to TD colon tissue collected in triplicate on three separate days. Figure S5: Average ultrafleXtreme MALDI IMS spectra from non-TD compared to TD ovary tissue collected in triplicate on three separate days. Figure S6: Average ultrafleXtreme MALDI IMS spectra from non-TD compared to TD pancreas tissue collected in triplicate on three separate days. Figure S7: Average ultrafleXtreme MALDI IMS spectra from non-TD compared to TD colon tissue. Figure S8: Average ultrafleXtreme MALDI IMS spectra from non-TD compared to TD ovary tissue. Figure S9: Average ultrafleXtreme MALDI IMS spectra from non-TD compared to TD pancreas tissue. Figure S10: MALDI FTICR IMS spectrum of peptide signal from nonthermally denatured and thermally denatured human colon, ovary, and pancreas tissue. Figure S11: MALDI IMS ion images, ion intensity plots, and post-MALDI IMS H&E stain of serial sections of nondenatured compared to thermally denatured human pancreas tissue. Figure S12: Comparison of pancreas tissue adherence after thermal denaturation using a regular indium tin oxide (ITO) slide compared to a poly lysine coated ITO slide. Figure S13: MALDI IMS ion images from nonthermally denatured compared to thermally denatured pancreas tissue. Figure S14: Average IMS spectra of region directly outside of non-TD compared to TD colon, ovary, and pancreas tissues (PDF)
Table S1: Table with identified and differentially abundant proteins from LC (XLSX)
Table S3: Proteins identified in LC-MS data from database search allowing for serine, threonine, tyrosine phosphorylation (XLSX)
Table S4: Proteins identified in LC-MS data from database search allowing for tyrosine sulfation (XLSX)
Table S5: List of MALDI features above a 5% intensity threshold in colon IMS with indication of ions detected in TD, NTD, or both tissue types along with every unthresholded feature in TD colon and NTD colon (XLSX)
Table S6: List of MALDI features above a 5% intensity threshold in ovary IMS with indication of ions detected in TD, NTD, or both tissue types along with every unthresholded feature in TD ovary and NTD ovary (XLSX)
Table S7: List of MALDI features above a 5% intensity threshold in pancreas IMS with indication of ions detected in TD, NTD, or both tissue types along with every unthresholded feature in TD pancreas and NTD pancreas (XLSX)
