# Supplementary material for: Thermal Denaturation of Fresh Frozen Tissue Enhances Mass Spectrometry Detection of Peptides
Source: Anal Chem. 2024 Oct 11;96(42):16861–70. doi: 10.1021/acs.analchem.4c03625 (PMC11503521; doi:10.1021/acs.analchem.4c03625)
Supplement: Supplementary file 1 — ac4c03625_si_001.pdf [file ac4c03625_si_001.pdf]

SUPPORTING INFORMATION

Thermal denaturation of fresh frozen tissue enhances mass spectrometry detection of peptides

Angela R.S. Kruse[±,○○,^], Audra M. Judd[±,ϕ,^], Danielle B. Gutierrez[±,ϕ], Jamie L. Allen[±,ϕ], Martin Dufresne[±,○○], Melissa A. Farrow[±,○○], Alvin C. Powers[ø,α], Jeremy L. Norris[±,±±,○], Richard M. Caprioli[±,ϕ,±±,**,++], Jeffrey M. Spraggins[±,ϕ,±±,○○*]

± Mass Spectrometry Research Center, Vanderbilt University, Nashville, Tennessee 37212, USA.

ϕDepartment of Biochemistry, Vanderbilt University, Nashville, Tennessee 37212, USA.

○○Department of Cell and Developmental Biology, Vanderbilt University, Nashville, Tennessee 37212, USA.

○Bruker Daltonics, Billerica 01821, Massachusetts, USA

øDepartment of Medicine, Division of Diabetes, Endocrinology, and Metabolism, Vanderbilt University School of Medicine, Nashville, Tennessee 37212, USA.

αVA Tennessee Valley Healthcare System, Nashville, Tennessee 37212, USA.

±±Department of Chemistry, Vanderbilt University, Nashville, Tennessee 37212, USA.

**Department of Medicine, Vanderbilt University, Nashville, Tennessee 37212, USA.

++Department of Pharmacology, Vanderbilt University, Nashville, Tennessee 37212, USA.

^Authors contributed equally
*Corresponding Author, jeff.spraggins@vanderbilt.edu

Table of Contents:

Table S2: PTXQC missed cleavage report
Figure S1. Amino acid composition of LC-MS identified peptides from microextraction samples from non-TD compared to TD tissues.
Figure S2: Identification of proteins containing phosphorylated serine, threonine, or tyrosine posttranslational modifications in colon and ovary.
Figure S3: Proteins with tyrosine sulfation detected in colon and ovary.
Figure S4. Average ultrafleXtreme MALDI IMS spectra from non-TD compared to TD colon tissue collected in triplicate on three separate days.
Figure S5. Average ultrafleXtreme MALDI IMS spectra from non-TD compared to TD ovary tissue collected in triplicate on three separate days.
Figure S6. Average ultrafleXtreme MALDI IMS spectra from non-TD compared to TD pancreas tissue collected in triplicate on three separate days.
Figure S7. Average ultrafleXtreme MALDI IMS spectra from non-TD compared to TD colon tissue.
Figure S8. Average ultrafleXtreme MALDI IMS spectra from non-TD compared to TD ovary tissue.
Figure S9. Average ultrafleXtreme MALDI IMS spectra from non-TD compared to TD pancreas tissue.
Figure S10. MALDI IMS spectrum of peptide signal from non-thermally denatured and thermally denatured human colon, ovary, and pancreas tissue.
Figure S11. MALDI IMS ion images, ion intensity plots, and post-MALDI IMS H&E stain of serial sections of non-denatured compared to thermally denatured human pancreas tissue.
Figure S12. Comparison of pancreas tissue adherence after thermal denaturation using a regular indium tin oxide (ITO) slide compared to a poly-lysine coated ITO slide.
Figure S13. MALDI IMS ion images from non-thermally denatured compared to thermally denatured pancreas tissue.
Figure S14. Average IMS spectra of region directly outside of non-TD compared to TD colon, ovary, and pancreas tissues.

Table S2: PTXQC missed cleavage report

Tissue	Average missed cleavages		p-value (T Test) (* p<0.05)
	Non-Thermally Denatured	Thermally Denatured	
Colon	0.49	0.39	0.07
Ovary	0.46	0.30	0.01*
Pancreas	0.40	0.31	0.16

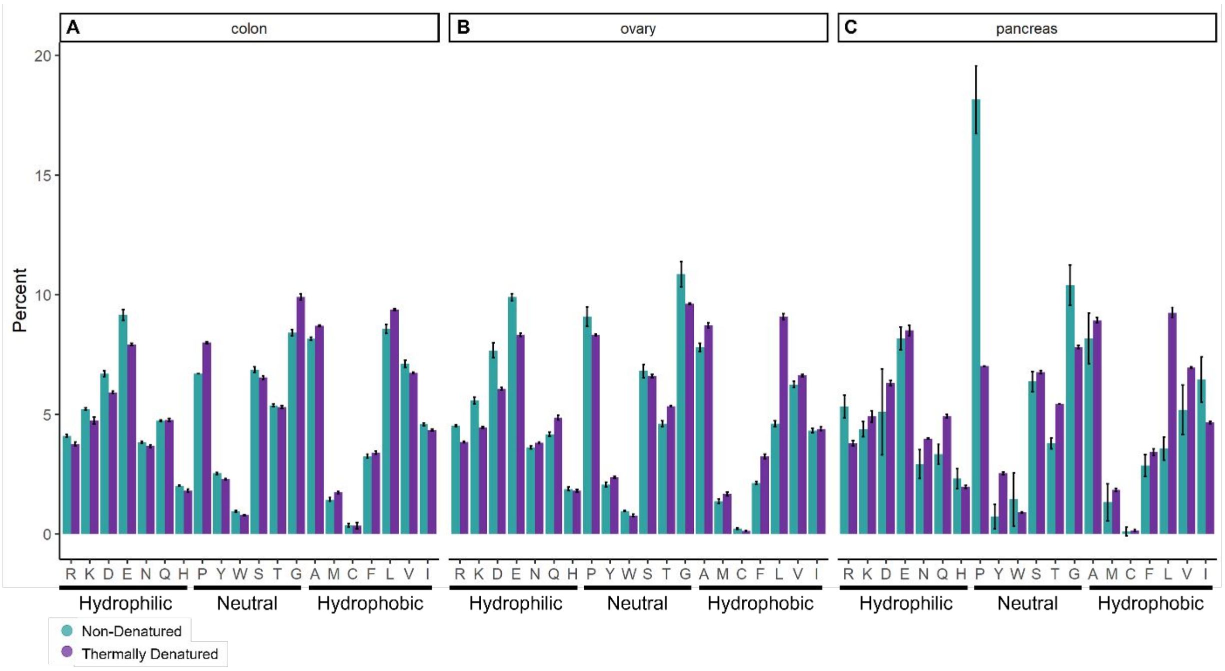

Figure S1. Amino acid composition of LC-MS identified peptides from microextraction samples from non-TD (blue) compared to TD (purple) tissues.

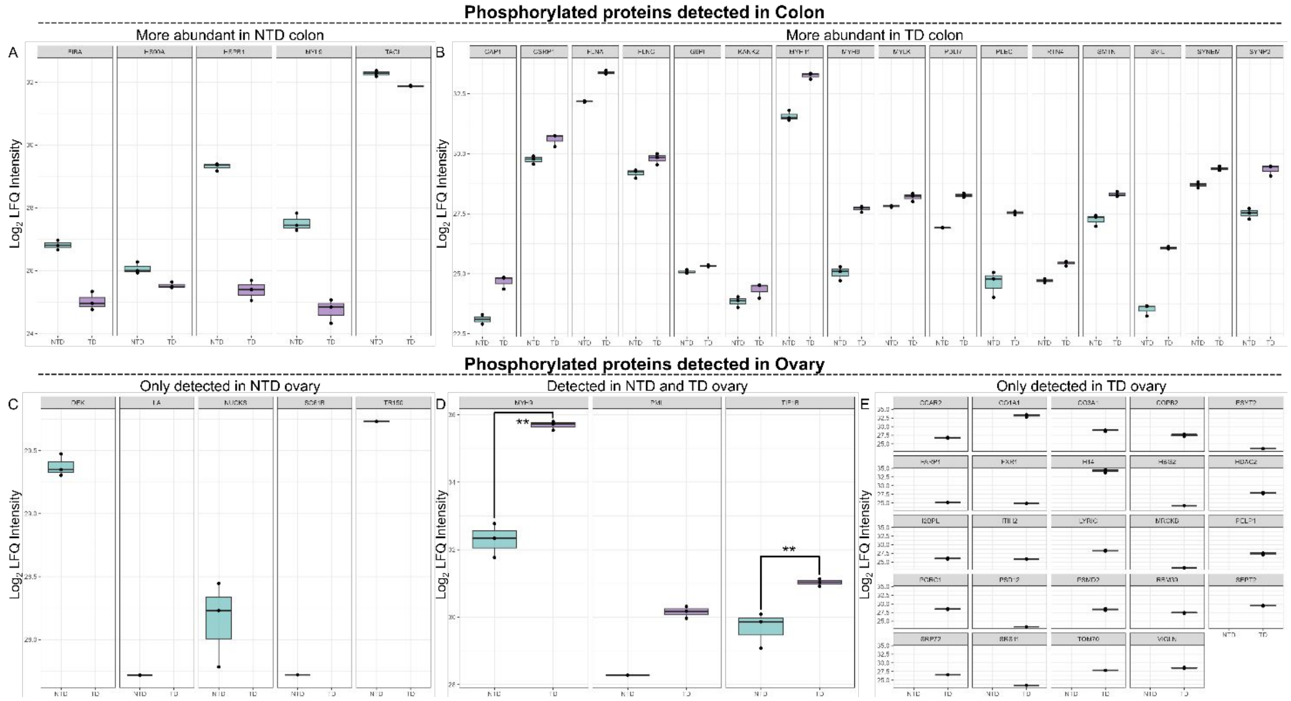

Figure S2. Identification of proteins containing phosphorylated serine, threonine, or tyrosine posttranslational modifications in colon (A-B) and ovary (C-E) tissue. In colon tissue, 21 of 85 identified phosphorylated were differentially abundant in thermally denatured (TD, purple) compared to non-thermally denatured (NTD, blue) samples. In ovary tissue, 2 of 32 phosphorylated proteins were differentially abundant in TD compared to NTD samples. A. Five phosphorylated proteins were statistically more abundant in NTD colon samples. B. Sixteen proteins were statistically more abundant in TD colon samples. C. Five proteins were only detected in NTD ovary samples. D. Three proteins were detected in both NTD and TD ovary samples, and two of these (MYH9 and TIF1B) had statistically higher abundance in TD ovary samples. ** indicates a p-value <0.01. E. Sixteen phosphorylated proteins were only detected in TD ovary samples. The $\log_2$ LFQ intensities for each sample were used to conduct a Welch's T test, and a P-value threshold of 0.05 was used to establish significance.

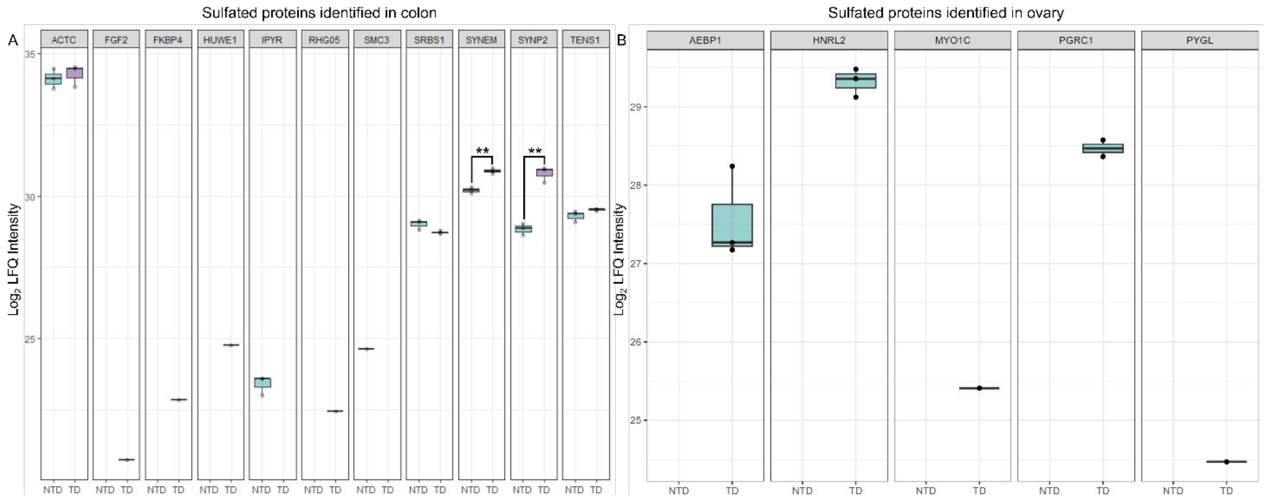

Figure S3. Proteins with tyrosine sulfation detected in colon and ovary tissue. A. In colon tissue, 11 proteins were identified with tyrosine sulfation. Of these, three (ACTC, SRBS1, and TENS1) were detected in both NTD (non-thermally denatured, blue) and TD (thermally denatured, purple) samples and were not differentially abundant. Five proteins were only detected in TD samples (FGF2, FKBP4, HUWE1, RHG05) and one was only detected in NTD samples (IPYR). Two had higher statistical abundance in TD samples (SYNEM, SYNP2) based on a Welch's T test, with a P-value threshold of 0.05. ** indicates a p-value <0.01. B. In ovary tissue, five sulfated proteins were detected, and all of these were only detected in TD tissues.

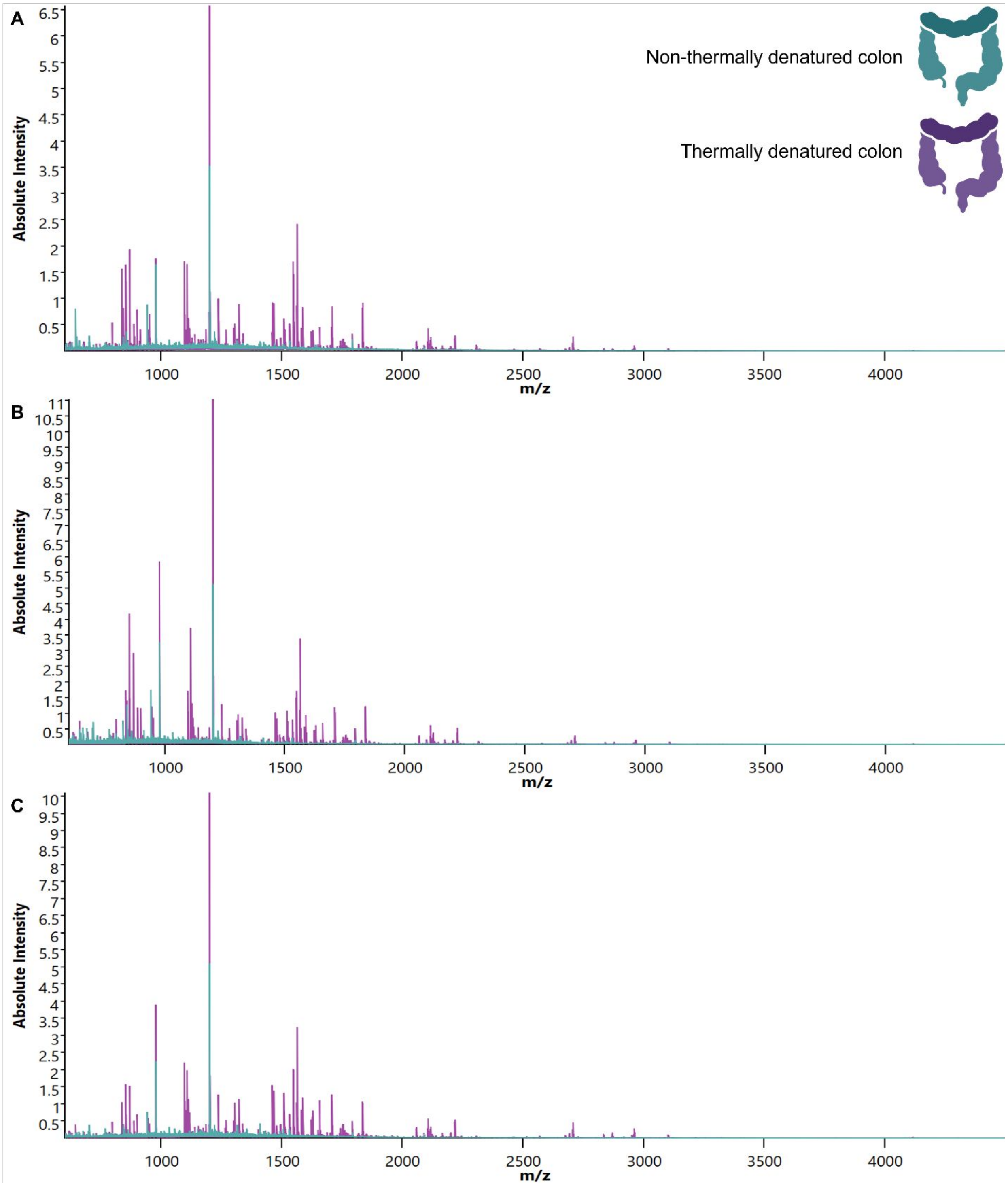

Figure S4. Average ultrafleXtreme MALDI IMS spectra from non-TD (blue) compared to TD (purple) colon tissue collected in triplicate on three separate days (shown in A,B,C)

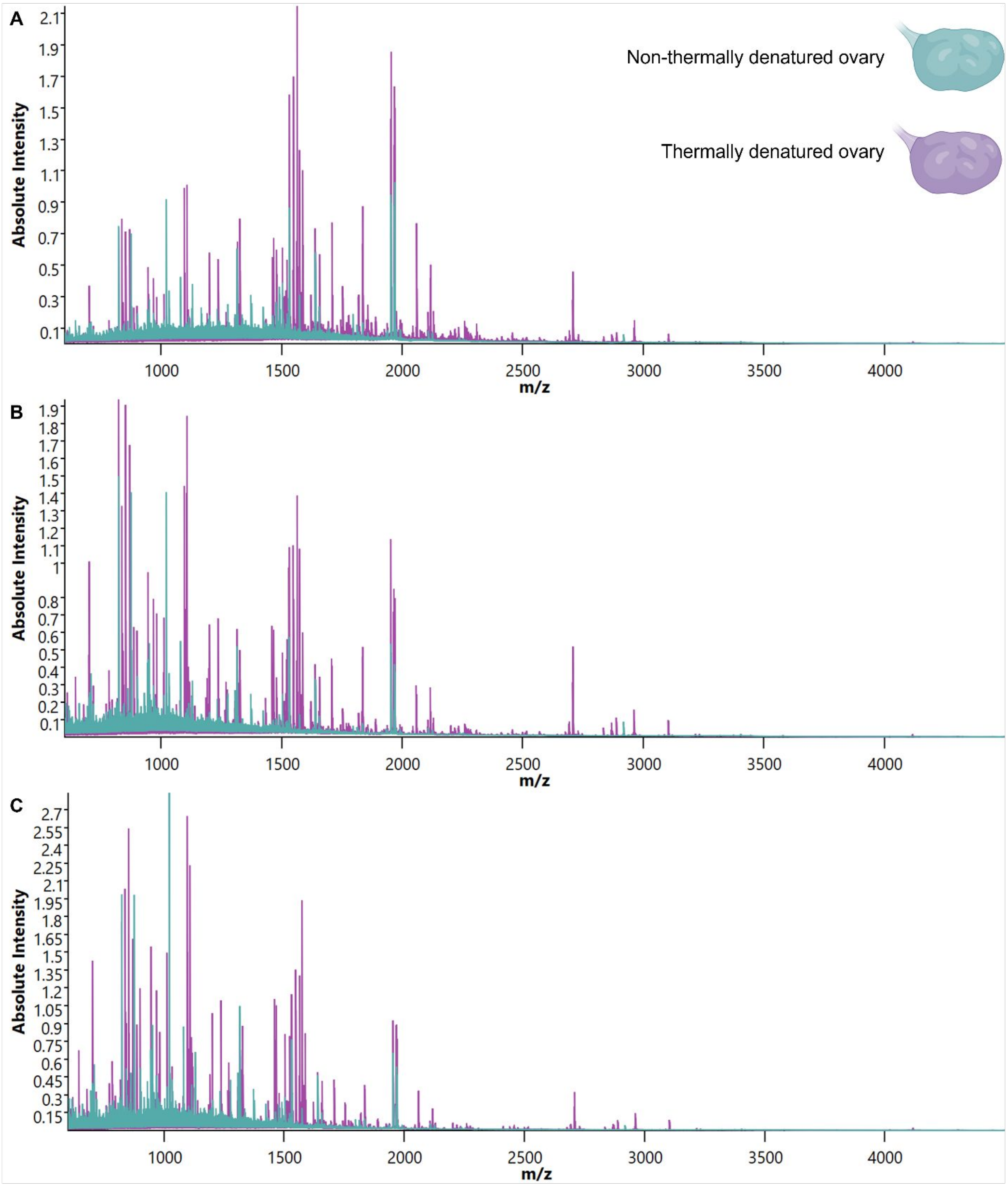

Figure S5. Average ultrafleXtreme MALDI IMS spectra from non-TD (blue) compared to TD (purple) ovary tissue collected in triplicate on three separate days (shown in A,B,C)

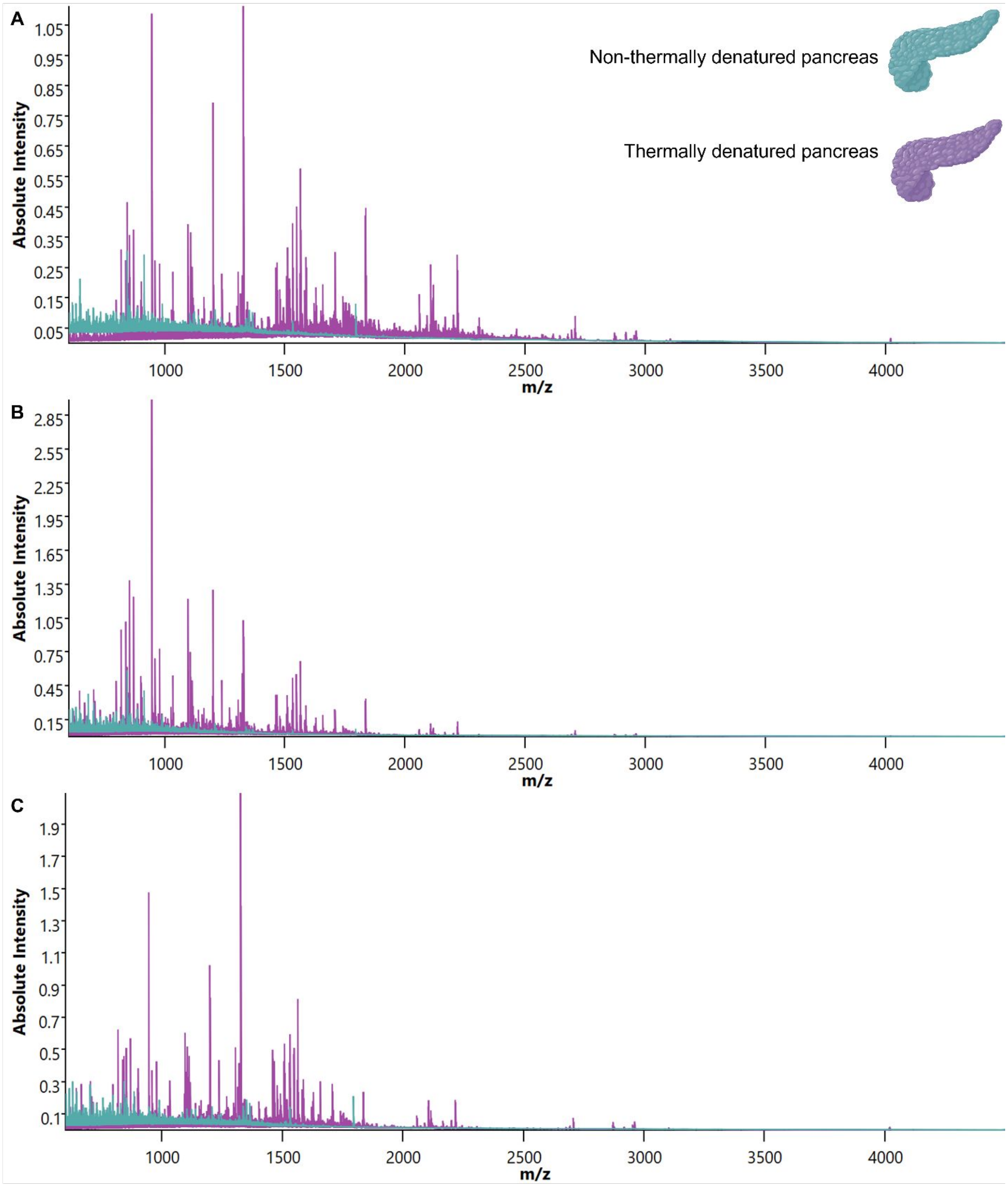

Figure S6. Average ultrafleXtreme MALDI IMS spectra from non-TD (blue) compared to TD (purple) pancreas tissue collected in triplicate on three separate days (shown in A,B,C)

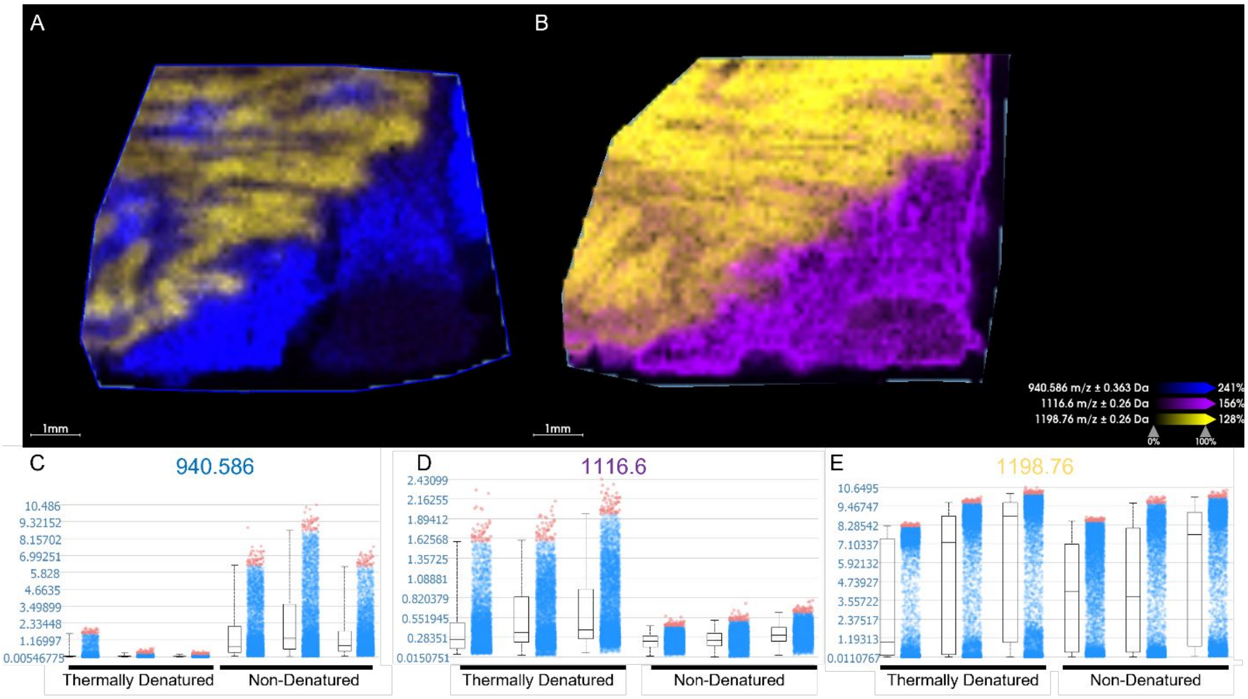

Figure S7. Average ultraflexXtreme MALDI IMS spectra from non-TD (A) compared to TD (B) colon tissue. Ion images (A,B) show comparable localizations of the ion at *m/z* 1198.76 (yellow) in both tissues (A,B,E), but *m/z* 940.586 (blue) is more abundant in non-TD colon (A, C) and *m/z* 1116.6 (purple) is more abundant in TD colon (B, D).

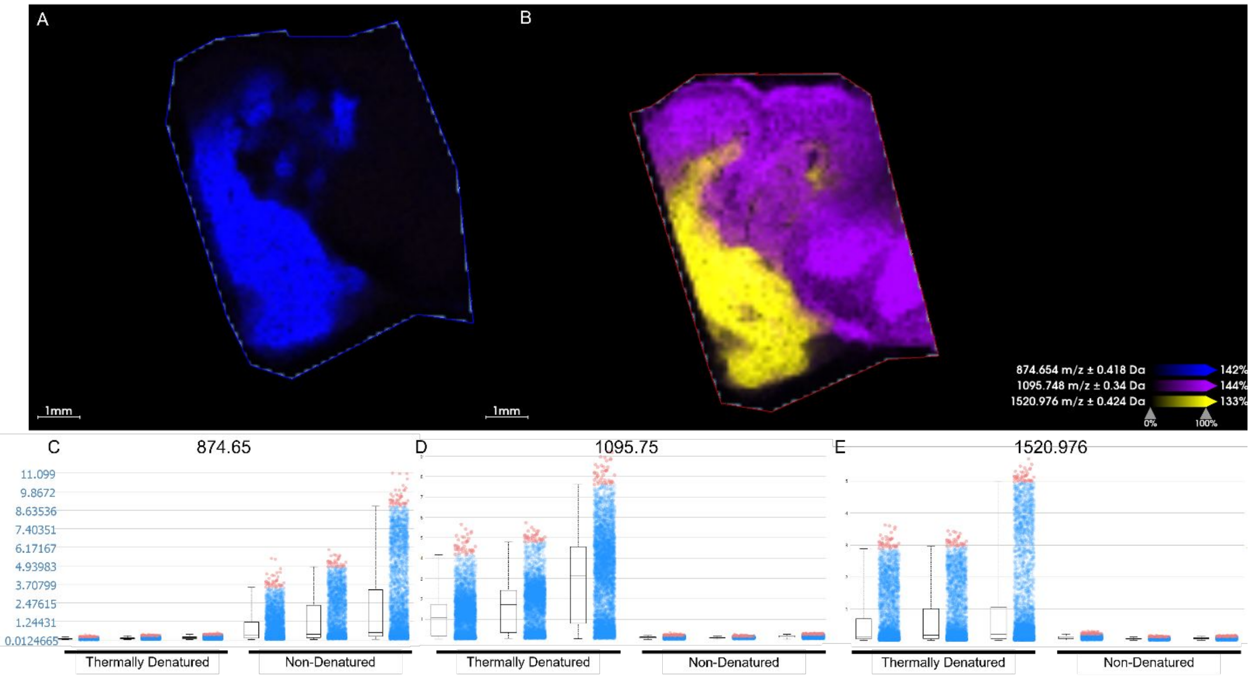

Figure S8. Average ultraflexXtreme MALDI IMS spectra from non-TD (A) compared to TD (B) ovary tissue. Ion images (A,B) show distinct localizations of the ions at *m/z* 874.654 (blue), 1095.748 (purple), and 1520.976 (yellow). *m/z* 874.654 (blue) is more abundant in non-TD ovary tissue (E) whereas *m/z's* 1095.748 (purple), and 1520.976 (yellow) are more abundant in TD ovary tissue (D,E).

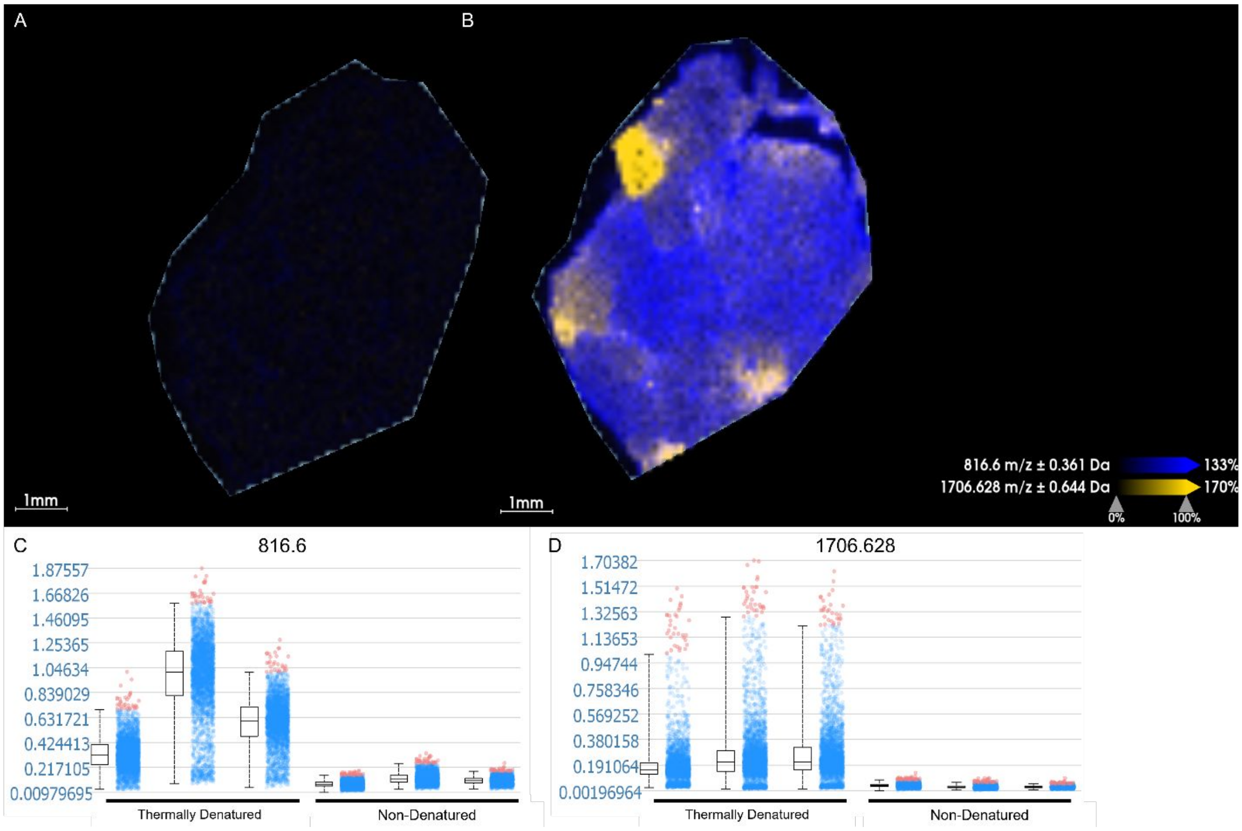

Figure S9. Average ultrafleXtreme MALDI IMS spectra from non-TD (A) compared to TD (B) pancreas tissue. Ion images (A,B) show distinct localizations of the ions at *m/z* 816.6 (blue), 1706.628 (yellow), which were more abundant in TD pancreas tissue (C,D).

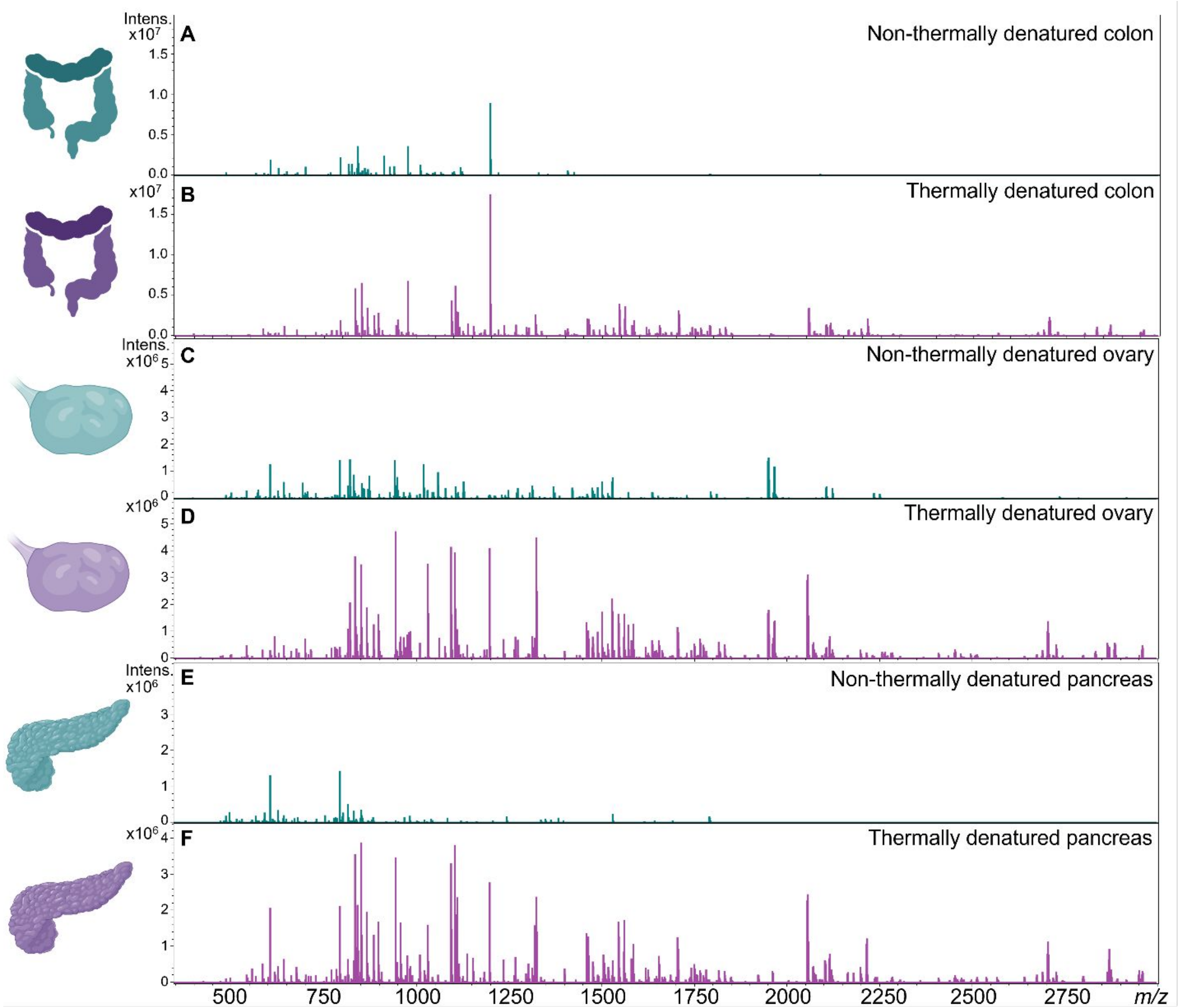

Figure S10. MALDI IMS spectrum of peptide signal from non-thermally denatured (blue) and thermally denatured (purple) human colon (A-B), ovary (C-D), and pancreas (E-F) tissue.

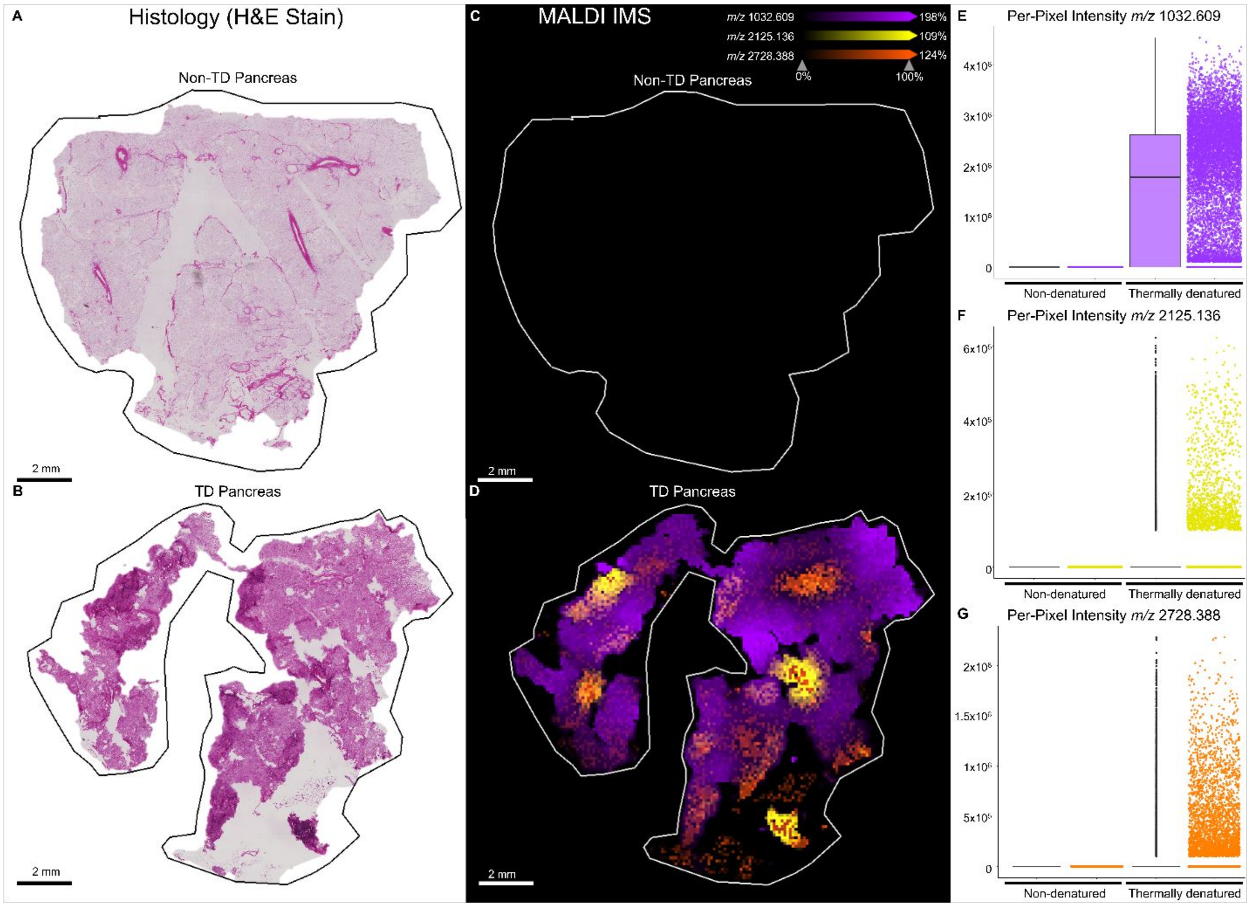

Figure S11. Post-MALDI IMS H&E stain of serial sections of non-denatured (A) compared to thermally denatured (B) human pancreas tissue. Ion images of peptides with *m/z* values 1032.6089 (purple), 2125.1356 (yellow), 2728.3882 (orange) in non-denatured (C) and TD (D) tissue. E, F, G. Ion intensity plots show dramatically increased detection of *m/z* 1032.6089, 2125.1356, and 2728.3882 in TD tissue (E, F, G, respectively).

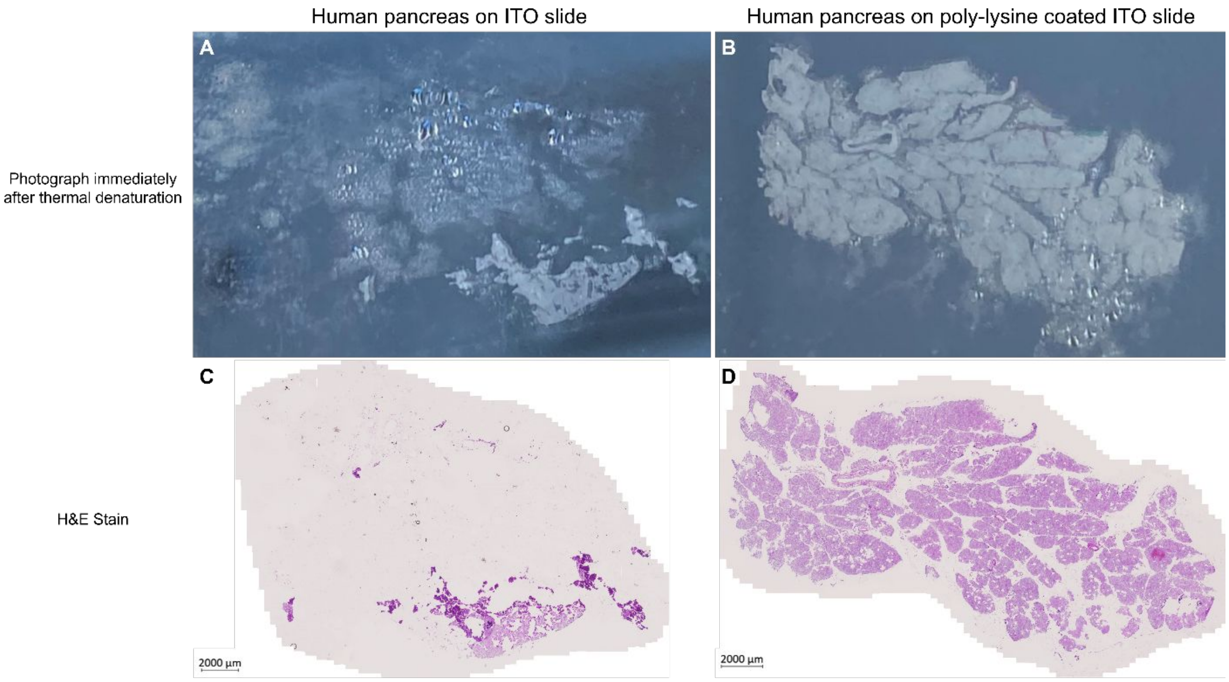

Figure S12. Comparison of pancreas tissue adherence using a regular indium tin oxide (ITO) slide (A, C) compared to a poly-lysine coated ITO slide (B,D). Serial pancreas tissue sections were photographed immediately after thermal denaturation showing very poor adherence to an ITO slide (A) and much improved adherence on a poly-lysine coated slide (B). Slides were then stained using H&E, demonstrating continued poor adherence to an ITO slide (C) and improved adherence to a poly-lysine coated ITO slide (D).

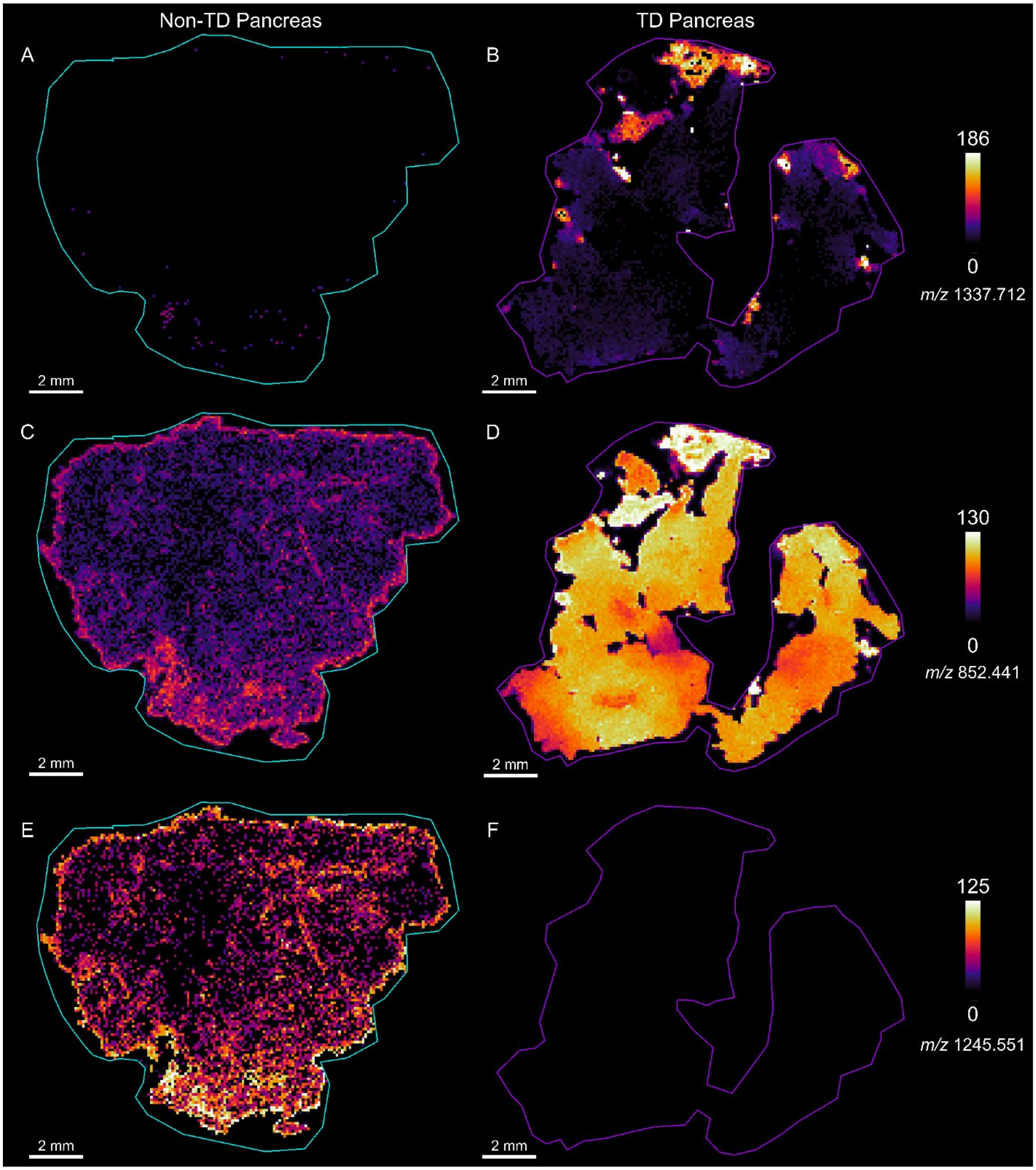

Figure S13. MALDI IMS ion images from non-thermally denatured (left) compared to thermally denatured (right) pancreas tissue.

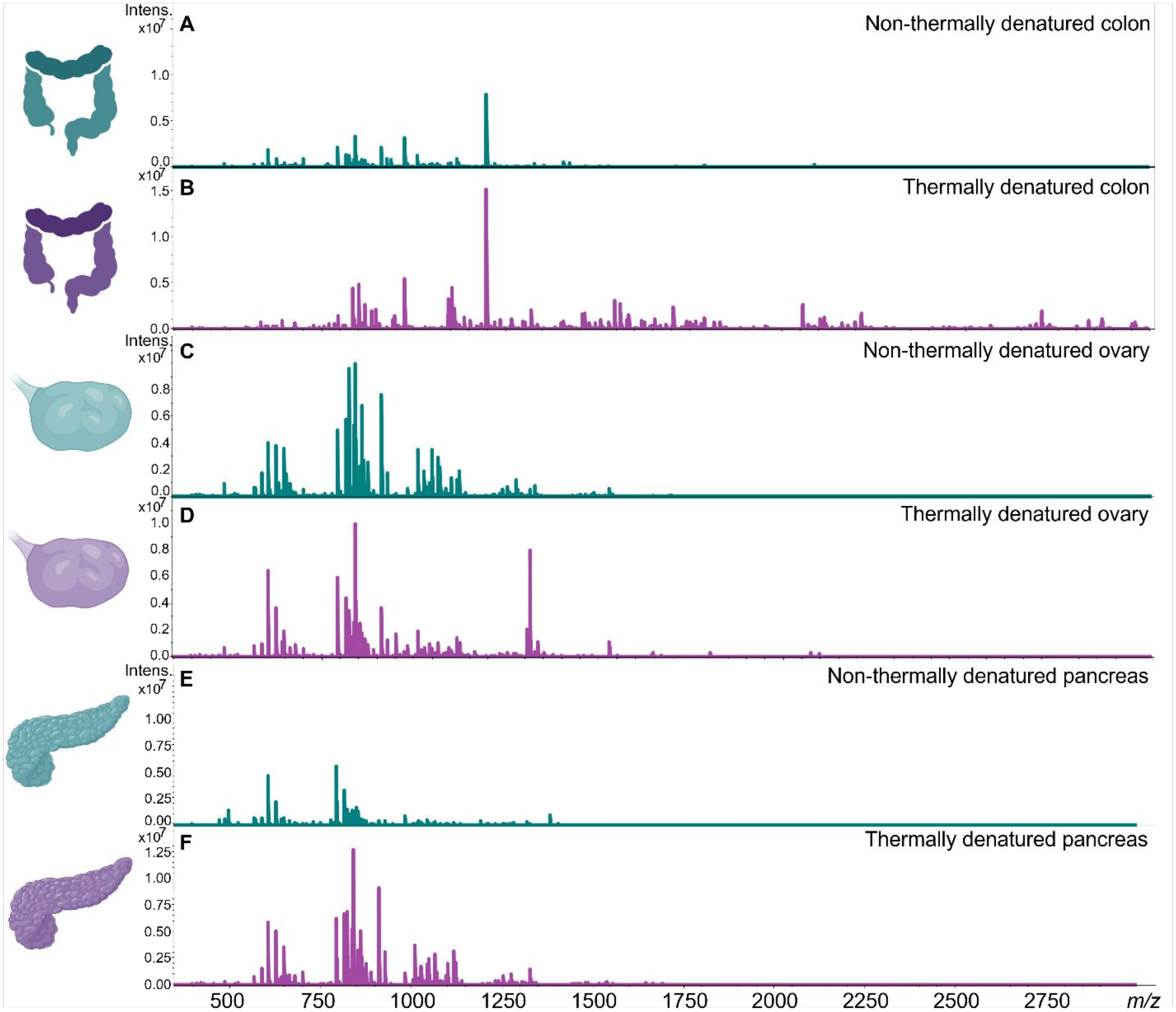

Figure S14. Average IMS spectra of the region directly outside of non-TD (blue) compared to TD (purple) colon, ovary, and pancreas tissues (A-B, C-D, E-F respectively).
